# Social norms and climate-friendly behavior of adolescents

**DOI:** 10.1371/journal.pone.0266847

**Published:** 2022-04-27

**Authors:** Ann-Kathrin Koessler, Tobias Vorlaufer, Florian Fiebelkorn

**Affiliations:** 1 Institute of Environmental Planning, Leibniz University Hannover, Hannover, Germany; 2 Alexander von Humboldt-Professorship of Environmental Economics, Institute of Environmental Systems Research and Faculty of Economics and Business Administration, Osnabrück, Germany; 3 Department of Geography and Environment, London School of Economics, London, United Kingdom; 4 Biology Didactics, Osnabrück University, Osnabrück, Germany; Polytechnic Institute of Coimbra: Instituto Politecnico de Coimbra, PORTUGAL

## Abstract

Adolescents are the decision-makers of the future, and as educational research shows, behaviors, habits, and attitudes established at young age strongly shape behavior in adulthood. Therefore, it is important to understand what factors shape young people’s climate-relevant behavior. In this study, we examine how information about peer behavior affects adolescents’ perception of prevailing social norms and own decision-making. Experimentally, we manipulated whether adolescents received information about other young people’s (lack of) support for climate protection, operationalized as a donation to a CO_2_ offsetting scheme. We find that empirical expectations shifted for all age groups when the information revealed that peers donated nothing or only small amounts. Donation behavior and the normative assessment, however, changed only in the younger age groups. Our study illustrates the caution that must be exercised when others’ behavior becomes visible or is deliberatively made salient in order to induce behavioral change, especially among young individuals.

## Introduction

Limiting global warming and its devastating consequences is a defining task of our time [1]. To secure the livelihoods of present and future generations, societies need to transform how they produce, consume, and live, which for many means renouncing their own resources and comfort [2–4]. Social science research suggests that shifting social norms is a promising approach to elicit the behavioral change required to achieve sustainability [5–7]. Social norms are behavioral rules that individuals prefer to follow when they believe that (i) sufficiently many others follow the rule (empirical expectation) and (ii) sufficiently many others believe one should follow it and may be willing to sanction deviations (normative expectation) [8]. Thus, a change in the perception of what behavior is common and considered socially desirable can encourage and reinforce climate-friendly behaviors.

A better understanding of the role of social norms in adolescent behavior can help to unlock the full transformative potential of social norms for climate action. First, simply due to their age, adolescents will contribute longer than older individuals to either climate change or its mitigation. Findings in developmental psychology and educational research suggest that values, attitudes, and habits formed during adolescence fundamentally determine behavior in later adulthood [9–11]. By engaging in climate protection today, young people are not only voicing the interests of future generations and influencing political decision-making, but also developing attitudes and values that will shape their own behavior as future decision-makers and citizens. Second, adolescents are generally considered particularly susceptible to the influence of social norms. Their identities and corresponding preferences are still developing [12, 13]; hence, adolescents search for cues as to appropriate behavior [14], they seek social approval from their peers and try to fit in [15–18]. Educational research has discussed the role of social norms in combating harmful behaviors, such as bullying [19] or substance abuse [20]. However, social norms can also promote pro-environmental behavior. Non-experimental studies from environmental education show that in addition to parental influence [21, 22], the attitudes and actions of friends and schoolmates shape adolescents’ pro-environmental attitudes and behavior [23–27].

While social norms exert a strong influence on adolescents, little is known about how adolescents react to norm-based interventions that aim to promote climate-friendly behaviors. While empirical work from various behavioral strands provides evidence for the potential of social norms and respective norm-based interventions to induce socially desired behavior [28–30], the first studies point to the need for caution and show that norm-based interventions (e.g., those increasing the salience of a specific behavior) can also backfire [31–33]. However, these studies focus exclusively on adults. Developing solid knowledge of the target population [33] and its culture [34] is warranted, as is further research to understand when and how new information about the behavior of others influences empirical and/or normative expectations and under what conditions the corresponding changes impact behavior.

Our study expands the existing knowledge on the role of social norms in adolescents’ pro-environmental behavior in two ways. First, we systematically analyze the behavioral impact of both types of information adolescents may obtain about their peers, namely, their level of support of climate change mitigation. This understanding helps to better assess the opportunities and risks of norm-based interventions that aim to foster climate-friendly behavior among adolescents. Second, to our knowledge, we are the first to study the role of social norms on young people’s behavior in a controlled experimental setting.

Previous studies on this relationship are survey-based [21, 23, 24], and as a consequence, the social and personal norms elicited as well as the actual behavior were highly correlated. Experimental studies, in contrast, allow researchers to selectively and exogenously alter the normative or empirical expectations. Additionally, we rely on an incentivized behavioral measure (i.e., an actual donation to a CO_2_ offsetting scheme), whereas existing studies used self-reported behavior or behavioral intentions. Comparative studies have shown that non-incentivized, self-reported survey responses can be biased [35] and often do not correspond with real behavior [36, 37]. In our study, each decision has direct and real consequences; a donation to a CO_2_ offsetting scheme serves as our measure for climate-friendly behavior. This operationalization has been used successfully in adult studies [38–40]. For our adolescents, however, donating money is new, even though they have already some experience with financial decision-making. Consequently, the normative and empirical expectations are less clear than for everyday behaviors, and the adolescents will search for cues about what behavior is appropriate. This setting provides us with a suitable testbed to assess whether and how information about the behavior of others influences adolescents’ perception of social norms and subsequent decision-making.

### Social norms as a determinant of (pro-environmental) behavior

A growing body of experimental research examining the role of social norms in adult behavior has shown that information about others’ behavior can alter individual decision-making, both in general [41–44] and in the pro-environmental domain [7, 29, 45, 46]. Learning about the behavior of others allows individuals to draw inferences about the predominant social norm. Yet, the existing literature shows that receiving this information either by observation or via social norm nudges does not always lead to (the desired) behavioral change [32, 44, 47]. Thus far, three factors have been identified in the literature to determine the effectiveness of social norm interventions. First, and perhaps obvious, individuals need to notice the new information, so the salience of the information is key for its influence [29]. Second, social similarity has been shown to determine the extent to which individuals are influenced by information about other people’s behavior. The more similarities the individual shares with the reference group, the more relevant the information about their behavior becomes and the stronger its influence [41, 46, 48, 49]. Third, social norms are based on two relevant beliefs: (a) *empirical expectations*, or what behavior the individual believes that others engage in, and (b) *normative expectations*, whether the individual believes the behavior is considered by sufficiently many others to be socially appropriate [8, 50]. Please note, some other authors distinguish between descriptive norms (i.e., what people in a group normally do) and injunctive norms (i.e., what people in a group deem to be appropriate behavior; e.g., [29, 51]). For the empirical work in this study, we chose to work with Bicchieri’s concept [8], as it clearly defines that the behavioral influence stems from social expectations.

The strongest influence of social norms on behavior is observed when both expectations coincide. When empirical and normative expectations are non-congruent, on the other hand, people tend to follow the empirical expectation, particularly when the observed behavior is in line with self-regarding preferences [41, 43]. Some studies suggest that observing others’ behavior may also update one’s normative expectations [44, 51]. However, this link is less established.

Participants in the aforementioned studies were adults, often university students. Little is known about the validity of the studies’ findings for other socio-demographic groups. In our study, we analyze whether and how 628 adolescents between 11 and 17 years of age change their empirical expectations, normative expectations, and, lastly, climate-friendly behavior in response to information they received about the behavior of relevant others. As a reference group, we chose peers with a high degree of socio-economic similarity (i.e., same age, same school type, and from the same city). Depending on the randomized treatment manipulation, participants received information that those relevant others donated either low or high amounts of their compensation for participation in the study to a CO_2_ offsetting scheme.

In the next sections, we describe our experimental design, hypotheses, and sample characteristics, followed by the experimental results section, before concluding with a discussion and avenues for future research.

## Experiment and hypotheses

The study was conducted in January 2020 at two high schools in Osnabrück, Germany with students from Grades 8, 9, and 10, who are typically between 13 and 16 years of age. Our approach of bringing the lab into schools has been successfully employed to probe the validity of findings from adult samples with regard to, for example, the rationality, risk, time, and social preferences of children and adolescents [52–54] and to learn about their development (for an overview, see [55]).

Approval for the study was obtained from the school principals and the state school authority. During school hours, students were invited to participate in a survey study on their attitudes toward climate protection. Participation in the survey was of course voluntary and the participation rate was 87%. As we have no information about the socio-economic characteristics of the non-participants, we cannot formally test whether a self-selection bias is present in our sample. Yet, given the relatively high participation rate, we consider potential self-selection effects to be limited. Students who did not want or who were not allowed by their parents to participate were supervised by their teachers in the meantime.

Students who participated received monetary compensation, which provided all students the same opportunity to donate. We obtained written informed consent for participation and anonymized data use from all participants. For students from Grades 8 and 9, we also obtained the written consent of one parent. A total of 628 adolescents participated, ranging in age from 11 to 17 (*Mean*: 14.5 years, *SD*: 1.04, *Median*: 15), and 55% were female.

In each school, the survey was conducted on one day during consecutive school lessons. All classes of the same grade, usually five classes with between 20 and 30 students each, met in the assembly hall during the designated lesson. After the students were welcomed, they received instructions on the process via a standardized presentation and learned that they would be compensated for their participation with 8 €. This corresponds to the average amount adolescents of this age group receive as weekly pocket money [56].

Students found the pen and paper surveys as prepared packages on spaced out chairs. The survey consisted of three parts, and students were asked to answer it in the pre-determined order. In the first part, students were informed that they could donate between 0 and 8 € of their compensation for participation for CO_2_ offsetting. The instructions described the NGO (*myclimate*) and what activities their donation would finance. The description also showed how students could monitor the transfer of the donation to the organization (we provided the donation receipts on a web page, listing all individual donation amounts with the corresponding participation code). The monitoring information was also used to implement our treatment, as described in the next section. Once students had made their donation decision, they were asked to pack this part in an envelope so that they could not return to it.

In the second part of the survey, students learned that they could earn one additional euro if they correctly guessed the average donation in their grade and school (belief elicitation 1). In the third part, students were asked to estimate how socially appropriate their schoolmates of the same grade rated each of the possible donation amounts on a four-point scale ranging from *very socially inappropriate* to *very socially appropriate* (belief elicitation 2). Since the concept of social appropriateness was for the younger age groups not trivial to grasp, we provided the following additional explanation: “By socially appropriate behavior, we mean behavior that is considered morally acceptable by the majority of people. If the majority is upset about a particular behavior of a person, then that behavior is considered socially inappropriate”. We tested the understanding of this concept during our pre-test.

Following the incentivized norm elicitation of Krupka and Weber [57], students could earn again one additional euro if they correctly guessed the most common answer given by their fellow students of the same grade. At the end of the study, we determined with a public lottery which donation amount from belief elicitation 2 was used to determine the payouts.

The two belief elicitations constitute our dependent variables empirical and normative expectations. At the end of the third part, students filled out a post-experimental questionnaire with questions on (i) their individual characteristics, such as age, gender, time at school, friends, neighborhood of residence, and languages spoken at home, as well as scales measuring the extent to which students’ engage in climate protecting behavior; (ii) their connectedness to nature (Inclusion of Nature in Self scale [58]); and (iii) whether they had participated in Fridays for Future protests in the past, in which pupils around the globe go on strike instead of going to school to campaign for better climate protection.

After all surveys were answered and collected, students received a debriefing, and during the next break or after school, they could come to payout stations in the assembly hall and collect their payout in a sealed envelope in exchange for their individual, but anonymous, participation code. This way it was ensured that also full anonymity and privacy of students’ (donation) decisions was maintained during the payment process. For students who could not pick up their payouts on the day of the survey, we provided additional opportunities to pick up their payouts in the following days. Due to the extensive pre-testing with open debriefings, the high degree of anonymity during filling-in the survey and pay-out process, and the voluntary consent that could be revoked at any time, we consider the overall (emotional) risks for participants as minimal.

### Treatments

The treatment manipulations consisted of only one text passage in the first part of the survey in which students learned how they could monitor the transfer of their donation to the NGO. In the treatment groups HIGH and LOW, three exemplary donations that were previously made by students of the same age from another anonymous high school from the same city were shown. In the LOW group, the three amounts ranged from 0 to 2 €, whereas in the HIGH group, the amounts ranged from 6 to 8 €. We pretested students’ understanding and perception of the survey at another local school and used this information to calibrate the amounts used for the endowment and incentivized belief elicitation. The donation amounts listed in the LOW and HIGH treatment were taken from this pretest.

Since our treatment intervention is based on the provision of donation amounts, these numbers may provide a reference point for the decision and thus trigger anchoring effects. Furthermore, providing information about donation amounts may induce experimenter-demand effects by giving conscious or subconscious cues about what donation we as experimenters may expect [59]. We capture these potential anchoring and demand effects with two additional treatment conditions, HYPO HIGH and HYPO LOW. In the HYPO conditions, the same high and low donation amounts were listed, but were explicitly labeled as hypothetical donations. Hence, demand effects may be even stronger in the two HYPO treatments than in the LOW and HIGH conditions, as the hypothetical nature of the listed donation amounts could be perceived by the respondents as more artificial than the actual donations listed in the HIGH and LOW treatments. Comparing the HIGH and LOW treatment effects with the respective HYPO treatments allows us to probe whether the treatment effects stem from the provision of information about peer behavior or are simply due to the provision of the numerical value and associated demand and/or anchoring effects. Since we considered these conditions for additional robustness analysis only, we assigned a smaller proportion of observations to these conditions in the random allocation. Lastly, in the CONTROL group, the instructions included information about how to monitor the transfer of each donation, but did not specify any exemplary donations.

Table 1 shows the treatment conditions and distribution of observations. Detailed summary statistics and tests probing the balance of our sample can be found in Section A in S1 File. Our random treatment allocation has resulted in a balanced sample, with no significant differences in observable socio-demographic characteristics across the treatment and control conditions. Students were randomly allocated to the five treatment conditions through the pre-packed survey packages laid out on the chairs in the schools’ assembly halls. The questionnaire versions differed only slightly on one page, and with students sitting far apart from each other (see Fig D.1–D.3 in S1 File) and being supervised by eight members of the research team, we can say with confidence that the students were not aware of the different versions during the study.

**Table 1:** Treatment overview.

Treatment	Description	Observations			
		Total	*Grade 8*	*Grade 9*	*Grade 10*
**CONTROL**	No information	164	51	53	60
**LOW**	Information on relevant others donating low amounts	159	49	50	60
**HIGH**	Information on relevant others donating high amounts	168	53	52	63
**HYPO-LOW**	Information on hypothetical low donation amounts	70	22	21	27
**HYPO-HIGH**	Information on hypothetical high donation amounts	67	22	19	26

The study was preregistered prior to data collection https://aspredicted.org/jz76g.pdf (Please note that the numbering of the hypotheses has changed). The dataset, analysis scripts, and original instructions (in German) can be accessed here: http://doi.org/10.17605/OSF.IO/Z6G54. An English translation of the instructions can be found in the Section D.2 in S1 File.

### Hypotheses

We expect that providing information about how much relevant others (i.e., students of the same age, school type, and city) chose to donate will alter students’ perceptions about the prevailing social norm and, consequently, behavior. We assessed these changes with the help of the two incentivized belief elicitations and the costly behavioral measure.

In belief elicitation 1, we targeted the **empirical expectation** and asked the students to guess how much their schoolmates of the same grade would donate on average. We hypothesized that this empirical expectation would be higher in HIGH, whose students received information about relevant others donating large amounts, in comparison to CONTROL (H1.1), in which no information about others’ donations was given. We expect the empirical expectation to be lower in the LOW condition compared to CONTROL (H1.2) and HIGH (H1.3).

With belief elicitation 2, we assessed the **normative expectations** and asked the students, following the mechanism of Krupka and Weber [57], to guess how socially appropriate their schoolmates of the same grade would perceive each possible donation amount. For the analysis, we will cluster the evaluations into small (0–2 €), medium (3–5 €), and high (6–8 €) donation amounts and will focus only on the evaluation of small and medium amounts, since we expect that high donation amounts will always be evaluated as socially desirable. (For the individual donations amounts in CONTROL, we observed that donations of 50% or more were judged on average as socially appropriate.*)*

We hypothesize that students will rate small and medium donation amounts lower on the social appropriateness scale in the HIGH condition than in CONTROL (H2.1). On the other hand, we hypothesize that the perceived social appropriateness of the same amounts will be higher in the LOW condition than in the CONTROL (H2.2) and HIGH (H2.3) conditions because the information that relevant others donated only little makes donating small amounts more socially acceptable.

Consequently, we assume that the information about the behavior of others also changes one’s own decision-making. Therefore, for observable behavior, we expect average donations to be higher in the HIGH condition compared to the CONTROL condition (H3.1) and lower in the LOW condition compared to the CONTROL (H3.2) and HIGH (H3.3) conditions.

## Results

In the following section, we examine the impact of the two information conditions, HIGH and LOW, first on the basis of aggregated data and one-sided Mann–Whitney U tests in the directions following our predictions.

**Fig 1:**
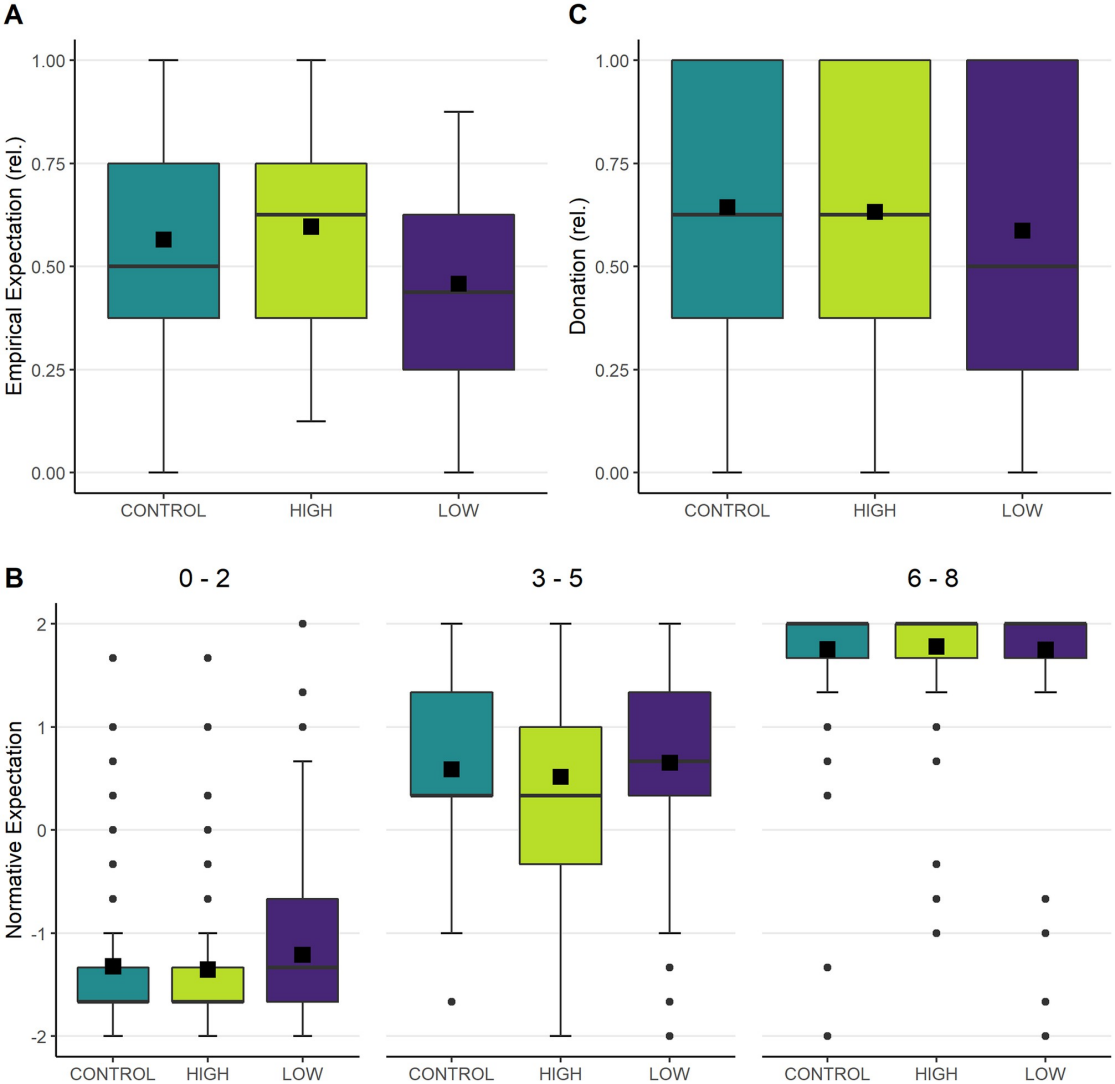
Evaluating the impact of the information about peers’ behavior. *Panel A* shows the distribution of the relative share of the endowment students thought their classmates would donate, on average. *Panel B* shows the rated social appropriateness for small (0–2 €), medium (3–5 €), and large (6–8 €) donation amounts for each condition. *Panel C* shows the distribution of donations. The data for all three measures is displayed as boxplots capturing the mean (square), the median (middle line), and the range of the relative donations (interquartile box–50% of data around the median, whiskers– 1.5 times the interquartile range).

**Panel A** in Fig 1 depicts the distribution of the **empirical expectations.** In the HIGH condition, students guessed that schoolmates of their grade would donate, on average, 60% of their endowment to CO_2_ offsetting. This donation is similar to what students in the CONTROL condition expected: 56% (*z* = -1.439, *p* = 0.075). Thus, we do not find support for H1.1. In the LOW group, in contrast, students believed that students of their grade would contribute, on average, 46% of their endowment; hence, they expected contribution of a significantly lower share than the CONTROL (*z* = 4.532, *p* < 0.001) and HIGH (*z* = -5.752, *p* < 0.001) groups did. We thus find support for H1.2 and H1.3: students altered their perception about the common behavior in response to the information provided, particularly when they received information that other students of the same age acted in a more self-regarding way.

A second element of social norms is the beliefs about what others think one should do: the **normative expectations**. To assess the social appropriateness of each donation amount, we clustered the evaluations into small (0–2 €), medium (3–5 €), and high (6–8 €) donation amounts. **Panel B** shows that *high donation* amounts were evaluated as very socially appropriate in all conditions. For *medium donation* amounts, the information that peers donated large amounts (HIGH) increased the variance, but did not alter the assessment in a significant manner (*z* = 0.542, *p* = 0.29). The information that peers donated low amounts (LOW), on the other hand, tended to increase the rated social appropriateness of medium amounts, but this change was also not statistically significant (comparison to CONTROL: *z* = -1.023, *p* = 0.153; comparison to HIGH: *z* = -1.472, *p* = 0.070). The assessment of *small donation* amounts, in contrast, was similar between students who received the HIGH information and the CONTROL group (*z* = -0.243, *p* = 0.596). When students instead obtained the information that peers donated low amounts (LOW), small donation amounts were judged as more socially appropriate. However, this observation was not statistically supported by the non-parametric tests (comparison with CONTROL: *z* = -1.307, *p* = 0.096; comparison with HIGH: *z* = -1.126, *p* = 0.130). Hence, we do not find support for H2.1, H2.2, and H2.3.

Lastly, we examined how the treatments influenced the **donation decisions** (**Panel C**). When students received the HIGH information, their donation decisions were, on average, similar to those by students in the CONTROL group (HIGH: 63% and CONTROL: 64% of the endowment; *z* = 0.273, *p* = 0.608). In the LOW condition, on the other hand, students showed a tendency to decrease their donations in response to the information about peers donating small amounts (LOW: 59% vs. CONTROL: 64%); however, no statistically significant difference in donation behavior can be detected compared to the CONTROL group (*z* = 1.365, *p* = 0.086) or the HIGH condition (*z* = 1.079, *p* = 0.140). Thus, we did not find support for H3.1, H3.2, and H3.3. Thus, in our setting, information about peers’ behavior has only a limited impact on the normative expectation and actual behavior on average. This finding is in line with the results of experimental research conducted with adults [44]; learning about the behavior of others does not automatically lead to a change in one’s own behavior.

**Fig 2:**
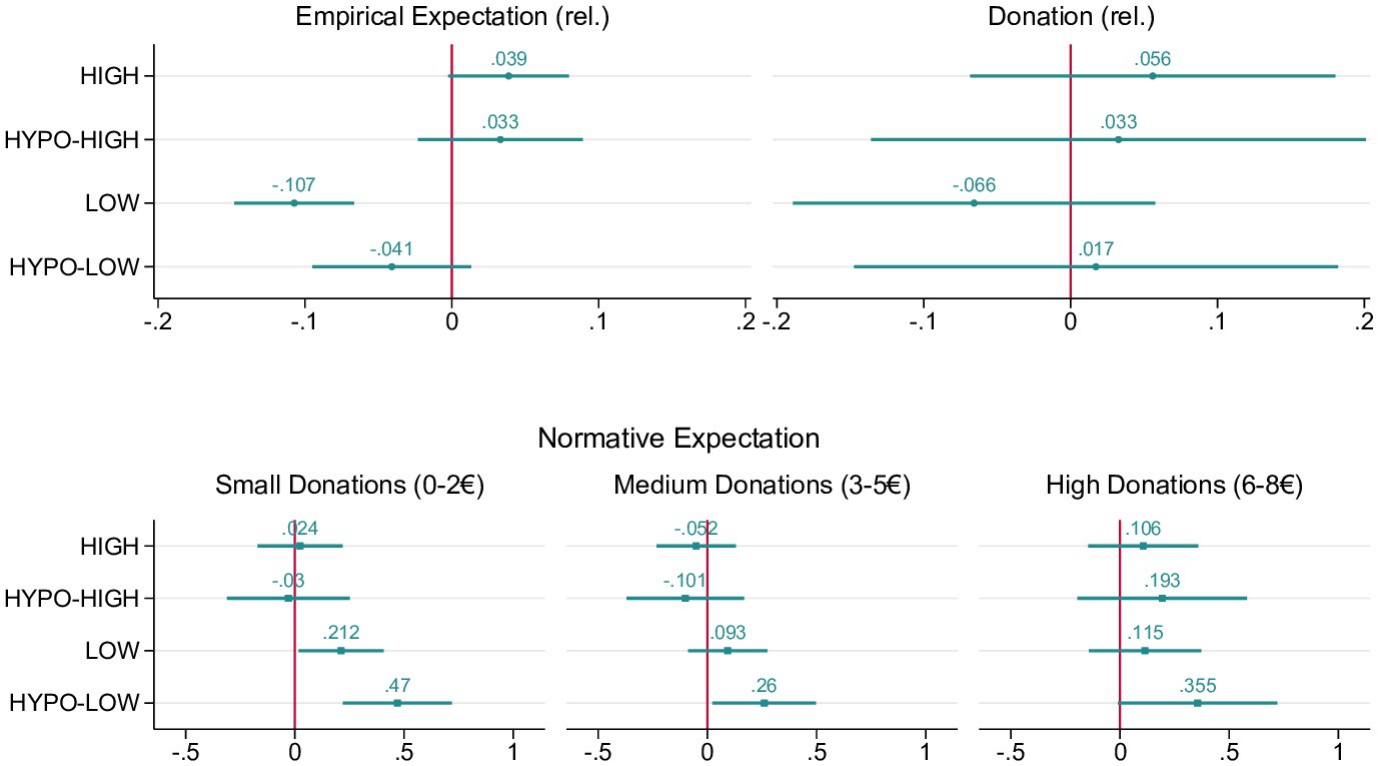
Coefficient plots of regression models estimating the treatment effects. This figure presents the tobit regression coefficients and estimated 95% confidence intervals. The coefficient interpretation is that if an individual was part of one of the four treatment conditions: HIGH, LOW, HYPO HIGH or HYPO LOW, then their expectations or donation amount is expected to change by the respective regression coefficient in the censored tobit scale, holding everything else constant. Full model results are reported in Section B in S1 File. Additional controls were gender, age, migration background, students’ connectedness to nature (Inclusion with Nature Scale [58]), existing engagement in climate-friendly behaviors, and participation in Fridays for Future protests. The models also include fixed effects on school class level.

To probe the robustness of our results, we performed **multivariate regression analyses** controlling for other factors that may have shaped students’ expectations and donation decisions, namely, individual characteristics, such as gender, age, migration background, students’ connectedness to nature (INS scale), existing engagement in climate-friendly behaviors (CFB scale, self-reported), participation on Fridays for Future protests, and effects of one’s school class. The models regress the answers for the empirical and normative expectations as well as the behavioral measure in the treatment conditions against the answers in CONTROL. The analyses were performed as tobit models, censored at the upper and lower limit of the donation scale, so that the estimations took into account that the possible donation amount was capped (see Section B.4 in S1 File). In addition, the regression models include the observations of our two HYPO conditions, in which the exemplary donation amounts were explicitly presented as hypothetical and fictitious. Anchoring and demand effects in HYPO should be at least as strong as in the LOW and HIGH conditions, so that the comparison will reveal whether the information describing peer behavior made a behavioral difference. In the following, we first describe the results from the treatments LOW and HIGH, and then the comparison to the HYPO conditions. Fig 2 plots the corresponding coefficients for LOW and HIGH as well as for HYPO LOW and HYPO HIGH. Alternative specifications without fixed effects on the school class level and with socio-demographic controls are reported in Section B.2–B.4 in S1 File.

For the empirical expectations (upper left), we replicate the results from the non-parametric analysis: only the treatment LOW has a significant effect on the empirical expectations (*p* <0.01, *CI*: [-0.148, -0.066]). While LOW dampens the empirical expectations, HIGH increases them albeit in a non-significant way, so that a significant difference is also observed between the two treatment conditions (LOW vs. HIGH, p = 0.000). For the normative expectations (bottom), in contrast to the non-parametric tests, we find that, for small donations (0–2 €), normative expectations in LOW are significantly different from those in CONTROL (*p* <0.05, *CI*: [0.017, 0.406]). This means that in the LOW condition students assumed their fellow schoolmates would consider small donations as more socially appropriate than students in CONTROL or HIGH (*p* = 0.059). Lastly, in the actual donations (upper right), the decreasing pull of LOW is visible, but the confidence intervals are generally wider [-0.189, 0.058] and the effect is not statistically significant.

Turning to the HYPO conditions, we find that providing hypothetical donation amounts as a reference shifted the empirical expectations only slightly. Unlike LOW, HYPO LOW does not alter students’ empirical expectations in a statistically significant manner. Thus, it made a significant difference whether the information was hypothetical or described peer behavior (LOW vs. HYPO LOW: *p* = 0.015). For the normative expectations, we find the reverse effect. When students were presented with small hypothetical donation amounts (HYPO LOW), they rated small and medium donation amounts as more socially appropriate. This was the case not only when compared to CONTROL, where no such information was available (*p* <0.01, *CI*: [0.220, 0.719] and *p* <0.05, *CI*: [0.023, 0.497], respectively), but also in comparison to the normative expectations in LOW, where the same amounts were presented but resembled donation decisions of peers (LOW vs. HYPO LOW: *p* = 0.041). We interpret this stronger shift as an indication for a more pronounced demand effect when reference donation amounts were hypothetical, we will come back to this point in the discussion. For the realized behavior, we find that the two HYPO conditions, HYPO LOW and HYPO HIGH, like their counterparts LOW and HIGH, do not unfold a significant effect on the giving decision. In summary, this means across all specifications we only find support for one of our predicted treatment effects: LOW decreases the empirical expectations relative to CONTROL and HIGH (H1.2 and H1.3).

### Age-specific analysis

**Fig 3:**
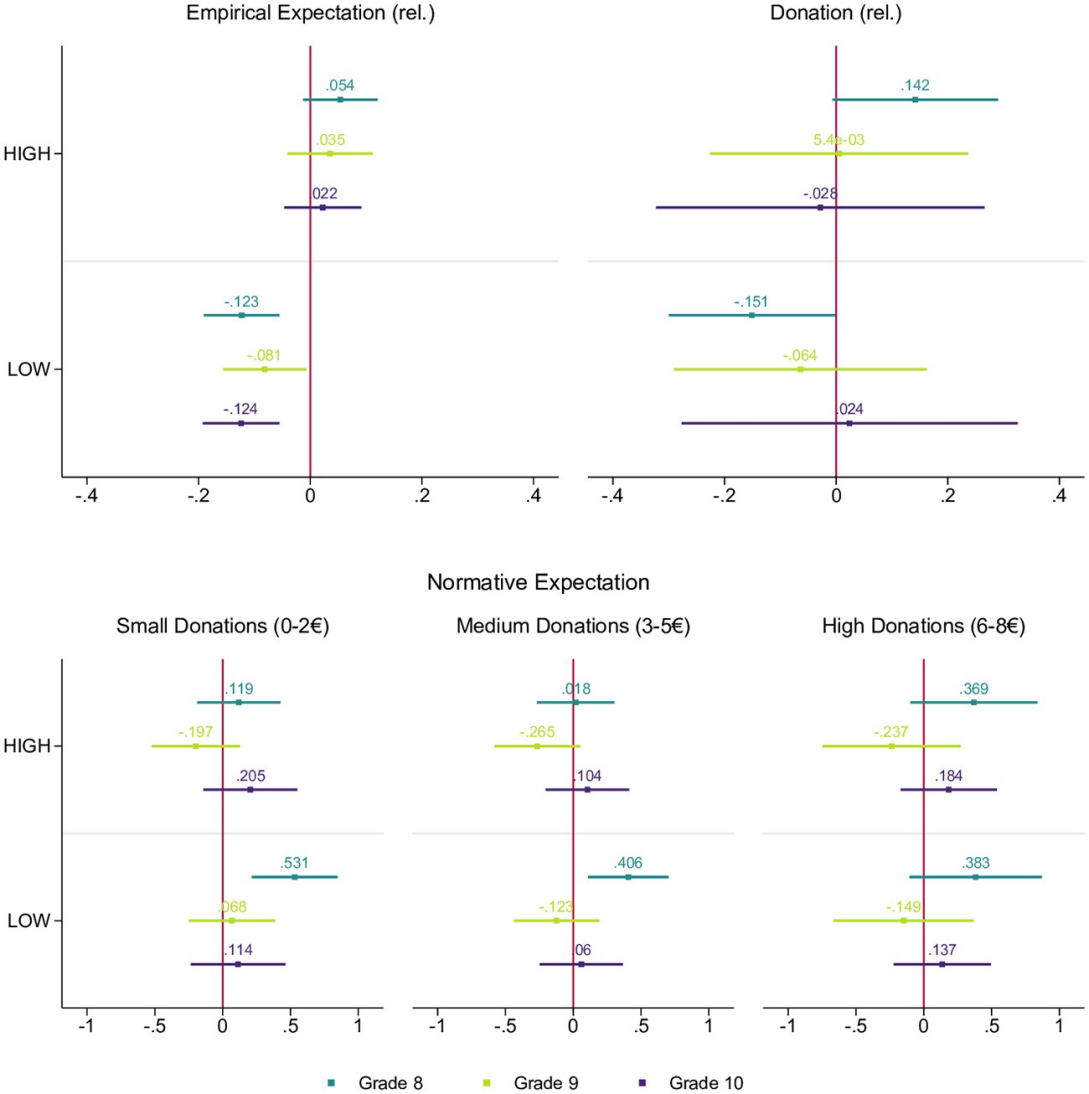
Grade-specific coefficient plots of regression models estimating the treatment effects. This figure presents the tobit regression coefficients and estimated 95% confidence intervals. The coefficient interpretation is that if a student in Grade 8, 9, or 10 was part of either the HIGH or LOW treatment, their donation amount is expected to change by the respective regression coefficient in the censored tobit scale, holding everything else constant. Full model results are reported in Section B.5 in S1 File. Additional controls were gender, age, migration background, students’ connectedness to nature (Inclusion with Nature Scale [58], existing engagement in climate-friendly behaviors, and participation on Fridays for Future protests. The models also include fixed effects on school class level.

To evaluate whether the influence of information about peers’ behavior is the same across all age groups, we performed an age-specific analysis by splitting the sample by grade. Fig 3 plots the corresponding coefficients, showing the treatment effects for Grade 8 (age 13/14, in medium turquoise), Grade 9 (age 14/15, in light green), and Grade 10 (age 15/16, in dark purple).

For the empirical expectations, we replicate the results from the aggregated analysis. The treatment information HIGH did not alter students’ empirical expectations, regardless of age, while LOW shifted the empirical expectations in all age groups (statistical significance: *p*<0.01 for Grades 8 and 10 and *p*<0.05 for Grade 9). For the normative expectations, similar to the overall analysis, we found that the treatment interventions did not influence students’ perception for Grades 9 and 10. However, the younger students in Grade 8 were significantly influenced by the information of the LOW condition and evaluated small and medium donation amounts as more socially appropriate than students in CONTROL did (*p*<0.1 for small and *p*<0.05 for medium donation amounts). The greater susceptibility of Grade 8 students is also reflected in their donation behavior: While the older students did not change their donation decision in response to the treatments, the eighth graders slightly increased their donations when treated with the HIGH information (*p*<0.1) and significantly reduced their donations when treated with the LOW condition (*p*<0.05).

We suggest two possible explanations for the age-specific effects of social norms: (i) those in older age groups adhere more to specific peers than to their general age group, and/or (ii) the older students have stronger established personal normative beliefs. Evidence supporting the latter includes the fact that self-reported climate-friendly behaviors are a strong predictor for the donation decision in Grade 10 and that participation on Fridays for Future protests was significantly more common in Grades 9 and 10 than in Grade 8 (p<0.01; see Table B.10 in S1 File). We will return to these points in the Discussion.

## Discussion and conclusion

In this study, we experimentally manipulated the information adolescents between 11 and 17 years old received about the (lack of) support of other young people for climate protection. We operationalized climate protection as a donation to a CO_2_ offsetting scheme and systematically assessed how information about previous low or high donation amounts made by peers of the same age influenced (i) adolescents’ beliefs about their fellow students’ donation behavior (empirical expectations), (ii) adolescents’ beliefs about how their fellow students ranked the social appropriateness of small and medium donation amounts (normative expectations), and, lastly, (iii) how the information influenced their own donation decision.

With regard to the different types of information, our study confirms a finding of previous social norm studies with adults: Individuals react more strongly based on information about others’ behavior when the described behavior is in line with one’s self-interest [43, 48]. In our case, this was the information that other students of the same age and from the same type of school and city had donated small amounts to climate protection. In response, the adolescents significantly lowered their expectations about how much their schoolmates of the same grade would donate. Beyond this, however, we found no effects on normative expectations and actual behavior. Contrary to our hypotheses, students, on average, did not alter their donation behavior in response to the treatment information.

One may argue that this is due to our subtle treatment manipulation—showing exemplary donations on a donation receipt is a relatively discreet way of conveying information about others’ behavior. However, we deliberately chose this subtle method to prevent students’ aligning their donation decisions to the information simply because they believe this is what we as experimenters expect of them. In addition to the general caution towards social desirability bias [60] and against inducing experimenter demand effects [59], it has been shown that children, in particular, have an initial tendency to rely on adults for social information [61, 62]. Thus, we believe that special caution is warranted when examining the effect of peer information on children’s and adolescents’ beliefs and behavior. The fact that empirical expectations shifted in response to the information about others’ low donations indicates that the adolescents in our sample did notice the treatment information. However, we can of course not rule out the possibility that a stronger intervention, with the drawbacks just mentioned, may also have triggered a behavioral change.

While the subtle treatment manipulation may explain the overall results, it does not explain our age-specific results. After all, the eighth graders reacted as predicted: they conformed to the observed behavior and donated less to the CO_2_ offsetting scheme. The older students in Grades 9 and 10 also changed their empirical expectations in response to the information that peers donated low amounts, but, in contrast to the younger students, this information did not affect their behavior.

We see two possible explanations for this age difference, which may also explain why, on the aggregate level, the peer information had only a limited effect. First, previous studies have shown that learning about the behavior of others is more effective when the reference group exhibits more similarities [43, 46, 48]. For example, Agerström et al. [63] provided university students with information about the donation behavior of either students from the same university or of students in Sweden in general. Only the information about the donation behavior of students from the same university increased average donations. To account for this determining factor, in our experiment, we chose a reference group that objectively shares many similarities with the surveyed adolescents; namely, they were the same age and from the same school type and city. However, it is possible that the chosen reference group was not specific enough for the adolescents, especially for the older students in Grades 9 and 10. After all, young people at this age are in the process of finding their own identity, and differentiation from the crowd is an essential part of this process. As a consequence, older adolescents may have a narrower reference group than younger ones. Our age-specific findings could align with this explanation: younger students in our sample considered students from another school (but the same age and city) as a reference group and thus updated their empirical expectations after receiving the information about these students’ donation decision and eventually adapted their own behavior. Older students, on the other hand, had a narrower reference group; while they also changed their empirical expectations, they did not change their behavior.

Another, possibly complementary, explanation is based on the stages of moral development. The ability to make principled moral judgments develops during adolescence [64, 65]. As a result, older adolescents are less guided by intrapersonal conformity; they reason more in terms of internalized norms and values. Applied to our study, this means that the older students likely already had stronger personal normative beliefs. This is indicated, for example, by the fact that they had a higher likelihood of having attended Fridays for Future protests, and that in the control group older students donated more than younger students. If the older students were more likely to form their donation decision on basis of their personal normative beliefs, then it is not surprising that the shift in empirical expectations did not suffice to influence their decision-making.

In sum, our findings suggest that the role of social norms for individual behavior is likely to change over the course of adolescence. Norm-based interventions can thus lead to mixed effects if not carefully designed for a specific age group. In particular, we observe that younger adolescents are more prone to reduce their climate-friendly behavior in response to the information that students from another school donated low amounts. Interventions aimed at making climate-related behavior more salient thus risk backfiring [32, 47], especially among younger students. At the same time, we observed that (younger) students did not increase donations after learning that others donated large amounts. This selective effect of social norms in one direction only might be related to the fact that climate-friendly behavior (i.e., donations to the CO_2_ offsetting scheme) was already relatively widespread at baseline; thus, the ability of social norms to induce socially desirable behavior may have been limited. Future research might aim to assess the effect of social norm interventions among adolescents for climate-friendly behaviors that are less common in the target group, such as consciously reducing meat consumption or data consumption on the internet. Findings from research on adults’ pro-environmental behavior [29, 45, 46] suggest that making the behavior of others salient under these circumstances can induce behavioral change toward the socially desirable behavior.

Further research is also warranted to better understand the change across age groups that we observed in our experimental data. We believe that the use of controlled experiments can help in this quest of gaining a deeper understanding of how social norms affect behavior over the course of adolescence and thus for the design of effective educational interventions [66]. In particular, we highly encourage future research to study a) whether reference groups for social norms change with age and b) whether the formation of personal normative beliefs dampens the influence of social norms on adolescent behavior with increasing age.

Though, we deem further research essential to provide a robust scientific foundation to develop recommendations for practitioners (in education, communication or public policy), we can outline some preliminary implications of our findings for practice. First, the overall effectiveness of social norm interventions seems limited among adolescents for (climate-friendly) behaviors that are already widespread. As suggested above, social norm intervention may bear greater potential for less common behaviors. Second, education programs and communication campaigns aimed at influencing youth behavior often target adolescents as a generic group. Our findings, however, underscore the importance of taking into account the fine-grained developmental stages during adolescence. Thus, inter- and transdisciplinary collaborations between behavioral and educational sciences and public policy are called for to understand adolescents’ perceptions and behavior, and to subsequently co-develop appropriate and effective interventions.

## Supporting information

S1 File (PDF)
