# Supplementary material for: Social norms and climate-friendly behavior of adolescents
Source: PLoS One. 2022 Apr 27;17(4):e0266847. doi: 10.1371/journal.pone.0266847 (PMC9045838; doi:10.1371/journal.pone.0266847)
Supplement: S1 File — (PDF) [file pone.0266847.s001.pdf]

Supporting Information

Social Norms and Pro-Environmental Behaviour of Adolescents

Ann-Kathrin Koessler, Tobias Vorlaufer & Florian Fielbekorn

Contents

A Socio-Demographic Characteristics by Treatment and Grade **2**

B Regression Model Results **7**
B.1 Coefficient Plots 7
B.2 Empirical Expectations 9
B.3 Normative Expectations 11
B.4 Donations 15
B.5 Treatment Effects by Grade 17

C Robustness Check: Hypo Treatments to control for Demand and Anchoring Effects **22**
C.1 Descriptives 22

D Experimental Material **24**
D.1 Pictures of the Implementation 24
D.2 Questionnaire and Instructions 26

A Socio-Demographic Characteristics by Treatment and Grade

In the following, we provide a brief overview of the covariates that are reported in the balancing tables and that are used in the regression analysis as controls:

Age (in years) Numeric

Female Dummy: 1 if gender is female, 0 otherwise

Foreign language spoken at home Dummy: 1 if language other than German is spoken at home, 0 otherwise

Most Close Friends in Same Class Dummy: 1 if most close friends are from the same class, 0 otherwise

Part. FFF protests Dummy: 1 if participated at least once in 2019 in Fridays-for-Future protests, 0 otherwise

INS Scale Numeric score between 1 and 7, answer to "How connected are you with nature?", a higher score indicates a closer connection with nature

CFB Scale Numeric score between 0 and 10, number of climate friendly behaviours listed, the following categories were included: "I am involved in the organization of the Fridays for Future-movement.", "I eat little or no meat.", "I try not to fly with the plane on vacations and also try to convince my parents not to do so either.", "I try to avoid using electricity when it is not needed.", "I try not to take long showers and not so often.", "I try to ride my bike often.", "I try to get my parents to buy more seasonal and regional products.", "I try to talk my parents into buying energy-efficient household appliances.", "I am convincing my parents to switch to green energy.", "I try to avoid generating (plastic) garbage."

Subject paid Dummy: 1 if subject was paid out, 0 otherwise

Table A.1: Sample Characteristics

	Mean	SD	Median	Min	Max	N
Age (in years)	14.48	1.04	15	11	17	619
Female	0.55	0.50	1	0	1	618
Foreign language spoken at home	0.27	0.45	0	0	1	611
Most Close Friends in Same Class	0.66	0.47	1	0	1	613
Part. FFF protests	0.53	0.50	1	0	1	625
INS Scale	4.13	1.23	4	1	7	621
CFB Scale	3.78	1.82	4	0	10	627
Subject paid	0.79	0.41	1	0	1	628

Table A.2: Sample Characteristics by Grade

Variable	N	(1) 8 Mean/SE	N	(2) 9 Mean/SE	N	(3) 10 Mean/SE	(1)-(2)	T-test Difference (1)-(3)	(2)-(3)
Age (in years)	194	13.325	191	14.445	234	15.462	-1.120***	-2.137***	-1.017***
		(0.040)		(0.039)		(0.037)			
Female	193	0.549	192	0.547	233	0.554	0.002	-0.004	-0.007
		(0.036)		(0.036)		(0.033)			
Foreign language spoken at home	192	0.286	186	0.280	233	0.258	0.007	0.029	0.022
		(0.033)		(0.033)		(0.029)			
Most Close Friends in Same Class	192	0.646	189	0.704	232	0.634	-0.058	0.012	0.070
		(0.035)		(0.033)		(0.032)			
Part. FFF protests	196	0.413	194	0.552	235	0.621	-0.138***	-0.208***	-0.070
		(0.035)		(0.036)		(0.032)			
INS Scale	194	4.263	193	3.974	234	4.145	0.289**	0.118	-0.171
		(0.092)		(0.089)		(0.076)			
CFB Scale	197	3.548	195	3.826	235	3.936	-0.277	-0.388**	-0.111
		(0.124)		(0.129)		(0.123)			
Subject paid	197	0.893	195	0.805	236	0.699	0.088**	0.194***	0.106**
		(0.022)		(0.028)		(0.030)			
F-test of joint significance (F-stat)							53.911***	192.670***	41.506***
F-test, number of observations							362	408	406

Notes: The value displayed for t-tests are the differences in the means across the groups. The value displayed for F-tests are the F-statistics. ***, **, and * indicate significance at the 1, 5, and 10 percent critical level.

Table A.3: Balancing Table - Main Treatments

	(1) CONTROL		(2) HIGH		(3) LOW		T-test Difference		
Variable	N	Mean/SE	N	Mean/SE	N	Mean/SE	(1)-(2)	(1)-(3)	(2)-(3)
Age (in years)	161	14.466<br>(0.084)	164	14.567<br>(0.083)	158	14.430<br>(0.082)	-0.101	0.035	0.137
Female	161	0.540<br>(0.039)	164	0.555<br>(0.039)	159	0.560<br>(0.039)	-0.015	-0.019	-0.005
Foreign language spoken at home	158	0.247<br>(0.034)	164	0.329<br>(0.037)	157	0.268<br>(0.035)	-0.082	-0.021	0.062
Most Close Friends in Same Class	160	0.656<br>(0.038)	163	0.663<br>(0.037)	155	0.665<br>(0.038)	-0.006	-0.008	-0.002
Part. FFF protests	163	0.595<br>(0.039)	166	0.524<br>(0.039)	159	0.579<br>(0.039)	0.071	0.016	-0.055
INS Scale	162	4.191<br>(0.096)	164	4.000<br>(0.088)	159	4.145<br>(0.096)	0.191	0.047	-0.145
CFB Scale	163	3.748<br>(0.155)	168	3.655<br>(0.135)	159	3.994<br>(0.148)	0.094	-0.245	-0.339*
Subject paid	164	0.805<br>(0.031)	168	0.762<br>(0.033)	159	0.780<br>(0.033)	0.043	0.025	-0.018
F-test of joint significance (F-stat)							1.070	0.441	0.831
F-test, number of observations							307	302	309

Notes: The value displayed for t-tests are the differences in the means across the groups. The value displayed for F-tests are the F-statistics. ***, **, and * indicate significance at the 1, 5, and 10 percent critical level.

Table A.4: Balancing Table - LOW and HYPO-LOW

Variable	(1) LOW N	(1) LOW Mean/SE	(2) HYPO-LOW N	(2) HYPO-LOW Mean/SE	T-test Difference (1)-(2)
Age (in years)	158	14.430	70	14.471	-0.041
		(0.082)		(0.120)	
Female	159	0.560	68	0.559	0.001
		(0.039)		(0.061)	
Foreign language spoken at home	157	0.268	69	0.246	0.021
		(0.035)		(0.052)	
Most Close Friends in Same Class	155	0.665	69	0.667	-0.002
		(0.038)		(0.057)	
Part. FFF protests	159	0.579	70	0.386	0.193***
		(0.039)		(0.059)	
INS Scale	159	4.145	70	4.114	0.030
		(0.096)		(0.150)	
CFB Scale	159	3.994	70	3.600	0.394
		(0.148)		(0.205)	
Subject paid	159	0.780	70	0.843	-0.063
		(0.033)		(0.044)	
F-test of joint significance (F-stat)					1.384
F-test, number of observations					219

Notes: The value displayed for t-tests are the differences in the means across the groups. The value displayed for F-tests are the F-statistics. ***, **, and * indicate significance at the 1, 5, and 10 percent critical level.

Table A.5: Balancing Table - HIGH and HYPO-HIGH

Variable	(1) HIGH N	(1) HIGH Mean/SE	(2) HYPO-HIGH N	(2) HYPO-HIGH Mean/SE	T-test Difference (1)-(2)
Age (in years)	164	14.567	66	14.409	0.158
		(0.083)		(0.128)	
Female	164	0.555	66	0.530	0.025
		(0.039)		(0.062)	
Foreign language spoken at home	164	0.329	63	0.238	0.091
		(0.037)		(0.054)	
Most Close Friends in Same Class	163	0.663	66	0.636	0.026
		(0.037)		(0.060)	
Part. FFF protests	166	0.524	67	0.463	0.061
		(0.039)		(0.061)	
INS Scale	164	4.000	66	4.273	-0.273
		(0.088)		(0.180)	
CFB Scale	168	3.655	67	3.851	-0.196
		(0.135)		(0.193)	
Subject paid	168	0.762	67	0.821	-0.059
		(0.033)		(0.047)	
F-test of joint significance (F-stat)					0.892
F-test, number of observations					219

Notes: The value displayed for t-tests are the differences in the means across the groups. The value displayed for F-tests are the F-statistics. ***, **, and * indicate significance at the 1, 5, and 10 percent critical level.

B Regression Model Results

B.1 Coefficient Plots

In the main article, regression results are reported through coefficient plots (see Figure B.1 and B.2). The respective regression results are reported within the regression tables in this chapter. Figure B.1 is based on the second models (2) in Table B.1 - B.5. Figure B.2 is based on all models presented in Table B.6 - B.10.

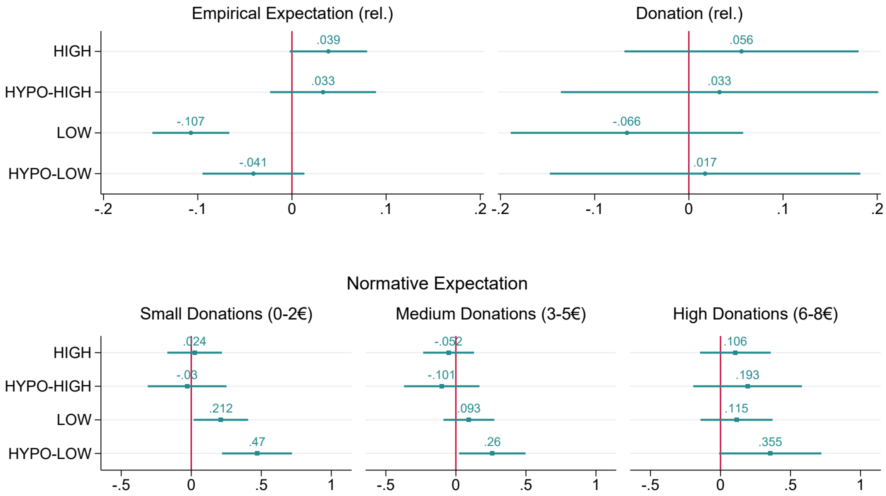

Figure B.1: Treatment Effect Estimates from Regression Models with 95% Confidence Intervals

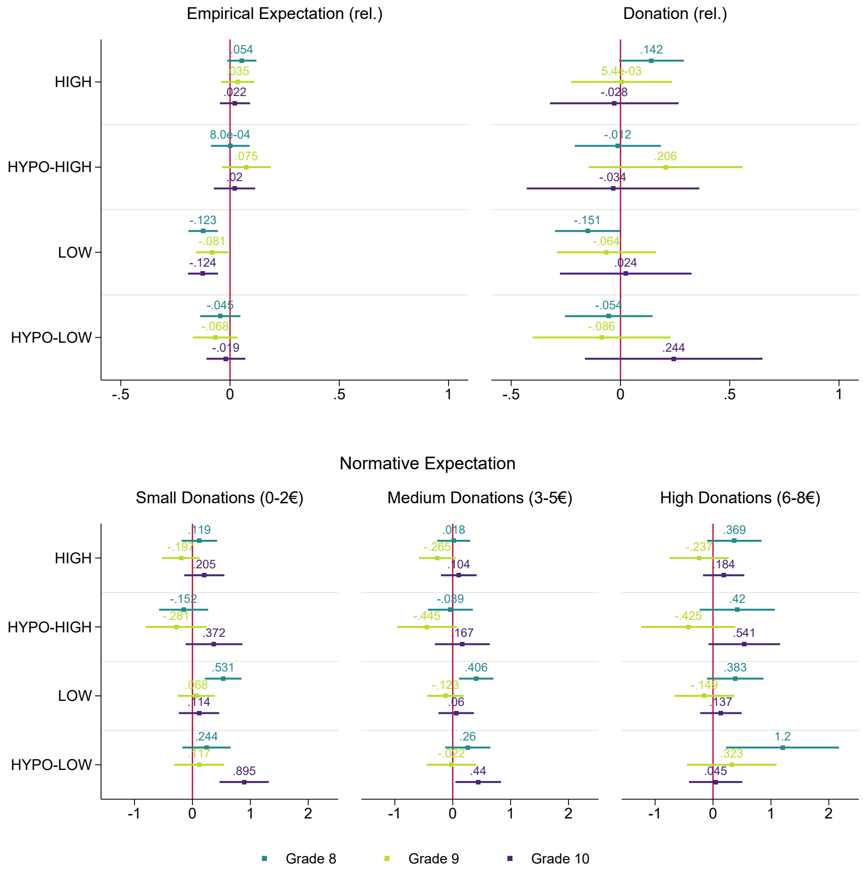

Figure B.2: Treatment Effect Estimates by Grade from Regression Models with 95% Confidence Intervals

B.2 Empirical Expectations

Regression analyses are performed as Tobit models, censored at the upper and lower limit of the scale for the belief elicitation 1 (0 and 1 as the share of the endowment of 8€ that could be donated). Control variables are age, gender, foreign language spoken at home, school, environmental attitudes (INS: Inclusion of Nature in Self scale), participation in Fridays for Future protests (dummy), climate-friendly behaviour (number of activities listed). Different specifications are provided that control a) for school fixed effects, b) class fixed effects and c) class fixed effects with socio-demographic controls only.

Table B.1: Regression Results - Empirical Expectation

	(1)	(2)	(3)
HIGH	0.0384*	0.0387*	0.0410**
	[-0.006,0.083]	[-0.002,0.080]	[0.000,0.082]
LOW	-0.110***	-0.107***	-0.106***
	[-0.154,-0.066]	[-0.148,-0.066]	[-0.147,-0.065]
HYPO-LOW	-0.0431	-0.0409	-0.0438
	[-0.100,0.014]	[-0.095,0.013]	[-0.097,0.010]
HYPO-HIGH	0.0123	0.0331	0.0296
	[-0.046,0.071]	[-0.023,0.089]	[-0.026,0.085]
Age (in years)	0.0417***	0.00356	0.00508
	[0.026,0.057]	[-0.024,0.031]	[-0.022,0.032]
Female	0.0702***	0.0658***	0.0682***
	[0.038,0.103]	[0.034,0.097]	[0.037,0.099]
For. Language: Yes	-0.0678***	-0.0564***	-0.0592***
	[-0.104,-0.031]	[-0.092,-0.021]	[-0.094,-0.024]
School B	-0.0912***		
	[-0.124,-0.059]		
INS Scale	-0.00291	-0.00377	
	[-0.017,0.011]	[-0.017,0.010]	
Part. FFF protests	0.0141	0.00515	
	[-0.019,0.048]	[-0.029,0.039]	
CFB Scale	0.00588	0.00432	
	[-0.004,0.015]	[-0.005,0.013]	
Constant	-0.0367	0.564**	0.543**
	[-0.269,0.196]	[0.124,1.003]	[0.110,0.975]
var(e.belief_rel)	0.0383***	0.0323***	0.0324***
	[0.034,0.043]	[0.029,0.036]	[0.029,0.036]
Class FE	No	Yes	Yes
N	592	587	591
p: HIGH = LOW	0.000	0.000	0.000
p: HYPO LOW = LOW	0.021	0.015	0.022
p: HYPO HIGH = HIGH	0.379	0.843	0.686

95% confidence intervals in brackets

* $p < 0.10$, ** $p < 0.05$, *** $p < 0.01$

B.3 Normative Expectations

To analyse how treatments affected the Normative Expectations of respondents, we compute the average social appropriateness scale for three donations categories: Small Donations (0-2€), Medium Donations (3-4€), and High Donations (6-8€). The initial responses were coded as follows:

- -2: Very socially inappropriate
- -1: Somewhat socially inappropriate
- 1: Somewhat socially appropriate
- 2: Very socially appropriate

As a result the average social appropriateness rating for each donation takes values between -2 and 2. The average social appropriateness rating for each possible donation amount in CONTROL is illustrated below in Figure B.3.

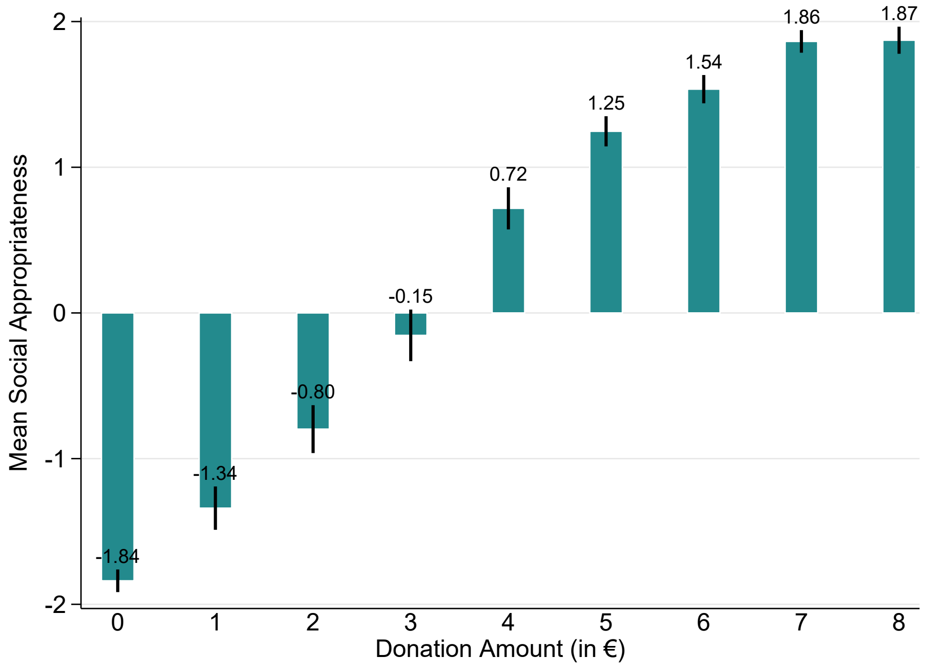

Figure B.3: Average Social Appropriateness Rating (Normative Expectation) for All Donation Amounts (CONTROL Treatment Only, Bars Indicate the 95% Confidence Interval)

Regression analyses are performed as Tobit models, censored at the upper and lower limit of the social appropriateness rating for the belief elicitation 2 (-2 and 2). Control variables are age, gender, foreign language spoken at home, school, environmental attitudes (INS: Inclusion of Nature in Self scale), participation in Fridays for Future protests (dummy), climate-friendly behaviour (number of activities listed). Different specifications are provided that control a) for school fixed effects, b) class fixed effects and c) class fixed effects with socio-demographic controls only.

Table B.2: Regression Results - Normative Expectation Small Donations (0-2€)

	(1)	(2)	(3)
HIGH	-0.0107	0.0237	0.0244
	[-0.211,0.190]	[-0.171,0.219]	[-0.170,0.219]
LOW	0.207**	0.212**	0.205**
	[0.005,0.408]	[0.017,0.406]	[0.011,0.399]
HYPO-LOW	0.463***	0.470***	0.457***
	[0.209,0.718]	[0.220,0.719]	[0.209,0.705]
HYPO-HIGH	0.0422	-0.0295	-0.0442
	[-0.237,0.321]	[-0.311,0.252]	[-0.322,0.234]
Age (in years)	-0.0402	0.0991	0.0917
	[-0.112,0.032]	[-0.031,0.229]	[-0.038,0.221]
Female	-0.325***	-0.317***	-0.325***
	[-0.473,-0.177]	[-0.466,-0.168]	[-0.473,-0.176]
For. Language: Yes	0.317***	0.298***	0.297***
	[0.149,0.485]	[0.130,0.467]	[0.130,0.465]
School B	0.0933		
	[-0.056,0.243]		
INS Scale	-0.0135	-0.0298	
	[-0.077,0.049]	[-0.093,0.034]	
Part. FFF protests	-0.0123	0.0754	
	[-0.167,0.143]	[-0.087,0.238]	
CFB Scale	-0.0295	-0.0158	
	[-0.074,0.015]	[-0.060,0.028]	
Constant	-0.654	-2.637**	-2.654**
	[-1.726,0.418]	[-4.703,-0.571]	[-4.708,-0.600]
var(e.sar02)	0.702***	0.634***	0.635***
	[0.605,0.798]	[0.547,0.721]	[0.548,0.723]
Class FE	No	Yes	Yes
N	543	539	540
p: HIGH = LOW	0.034	0.059	0.070
p: HYPO LOW = LOW	0.048	0.041	0.045
p: HYPO HIGH = HIGH	0.709	0.707	0.625

95% confidence intervals in brackets

* $p < 0.10$, ** $p < 0.05$, *** $p < 0.01$

Table B.3: Regression Results - Normative Expectation Medium Donations (3-5€)

	(1)	(2)	(3)
HIGH	-0.0655	-0.0517	-0.0594
	[-0.255,0.124]	[-0.233,0.130]	[-0.241,0.122]
LOW	0.108	0.0928	0.0836
	[-0.082,0.299]	[-0.089,0.275]	[-0.098,0.265]
HYPO-LOW	0.269**	0.260**	0.263**
	[0.025,0.514]	[0.023,0.497]	[0.027,0.500]
HYPO-HIGH	-0.00912	-0.101	-0.0994
	[-0.278,0.260]	[-0.371,0.169]	[-0.366,0.167]
Age (in years)	-0.0546	0.0647	0.0633
	[-0.123,0.014]	[-0.059,0.189]	[-0.060,0.187]
Female	-0.154**	-0.102	-0.103
	[-0.295,-0.014]	[-0.243,0.039]	[-0.244,0.037]
For. Language: Yes	0.228***	0.224***	0.228***
	[0.067,0.389]	[0.064,0.384]	[0.069,0.387]
School B	0.240***		
	[0.097,0.382]		
INS Scale	0.0520*	0.0371	
	[-0.008,0.112]	[-0.023,0.097]	
Part. FFF protests	-0.0305	0.00211	
	[-0.177,0.116]	[-0.151,0.156]	
CFB Scale	-0.0362*	-0.0254	
	[-0.078,0.006]	[-0.066,0.016]	
Constant	1.234**	-0.598	-0.518
	[0.209,2.259]	[-2.570,1.374]	[-2.481,1.446]
var(e.sar35)	0.650***	0.580***	0.581***
	[0.568,0.731]	[0.507,0.653]	[0.508,0.655]
Class FE	No	Yes	Yes
N	534	530	531
p: HIGH = LOW	0.072	0.121	0.125
p: HYPO LOW = LOW	0.198	0.166	0.135
p: HYPO HIGH = HIGH	0.681	0.719	0.768

95% confidence intervals in brackets

* $p < 0.10$, ** $p < 0.05$, *** $p < 0.01$

Table B.4: Regression Results - Normative Expectation High Donations (6-8€)

	(1)	(2)	(3)
HIGH	0.0730	0.106	0.101
	[-0.188,0.334]	[-0.146,0.359]	[-0.152,0.354]
LOW	0.0960	0.115	0.106
	[-0.169,0.361]	[-0.143,0.372]	[-0.151,0.364]
HYPO-LOW	0.396**	0.355*	0.369**
	[0.022,0.770]	[-0.010,0.721]	[0.004,0.734]
HYPO-HIGH	0.306	0.193	0.230
	[-0.080,0.691]	[-0.195,0.581]	[-0.157,0.617]
Age (in years)	-0.0364	0.0116	0.0105
	[-0.133,0.060]	[-0.165,0.188]	[-0.166,0.187]
Female	-0.0695	-0.0934	-0.0956
	[-0.268,0.129]	[-0.295,0.108]	[-0.297,0.106]
For. Language: Yes	0.121	0.106	0.119
	[-0.109,0.351]	[-0.128,0.340]	[-0.113,0.352]
School B	0.0830		
	[-0.119,0.284]		
INS Scale	0.0283	0.0360	
	[-0.058,0.115]	[-0.053,0.125]	
Part. FFF protests	-0.103	-0.0833	
	[-0.310,0.105]	[-0.302,0.135]	
CFB Scale	-0.0176	-0.0204	
	[-0.077,0.042]	[-0.079,0.038]	
Constant	2.744***	2.127	2.170
	[1.294,4.193]	[-0.695,4.949]	[-0.640,4.979]
var(e.sar68)	0.944***	0.845***	0.852***
	[0.738,1.149]	[0.660,1.030]	[0.666,1.038]
Class FE	No	Yes	Yes
N	537	533	534
p: HIGH = LOW	0.865	0.949	0.967
p: HYPO LOW = LOW	0.119	0.196	0.157
p: HYPO HIGH = HIGH	0.239	0.660	0.513

95% confidence intervals in brackets

* $p < 0.10$, ** $p < 0.05$, *** $p < 0.01$

B.4 Donations

Regression analyses are performed as Tobit models, censored at the upper and lower limit of the donation scale (0 and 1 as the share of the endowment of 8€ that was donated). Control variables are age, gender, foreign language spoken at home, school, environmental attitudes (INS: Inclusion of Nature in Self scale), participation in Fridays for Future protests (dummy), climate-friendly behaviour (number of activities listed). Different specifications are provided that control a) for school fixed effects, b) class fixed effects and c) class fixed effects with socio-demographic controls only.

Table B.5: Regression Results - Donations

	(1)	(2)	(3)
HIGH	0.0442	0.0559	0.0410
	[-0.086,0.174]	[-0.068,0.180]	[-0.085,0.167]
LOW	-0.0783	-0.0657	-0.0569
	[-0.208,0.051]	[-0.189,0.058]	[-0.182,0.068]
HYPO-LOW	-0.00186	0.0172	-0.00460
	[-0.170,0.167]	[-0.148,0.182]	[-0.171,0.162]
HYPO-HIGH	-0.0297	0.0326	0.0441
	[-0.200,0.141]	[-0.136,0.201]	[-0.126,0.215]
Age (in years)	0.111***	0.00859	0.00343
	[0.065,0.157]	[-0.075,0.092]	[-0.080,0.087]
Female	0.244***	0.235***	0.260***
	[0.148,0.340]	[0.140,0.330]	[0.164,0.356]
For. Language: Yes	-0.326***	-0.299***	-0.330***
	[-0.433,-0.220]	[-0.405,-0.194]	[-0.437,-0.224]
School B	-0.168***		
	[-0.263,-0.073]		
INS Scale	0.0339	0.0325	
	[-0.007,0.075]	[-0.008,0.073]	
Part. FFF protests	0.112**	0.0855*	
	[0.013,0.210]	[-0.016,0.187]	
CFB Scale	0.0554***	0.0538***	
	[0.027,0.084]	[0.026,0.081]	
Constant	-1.225***	0.409	0.849
	[-1.919,-0.532]	[-0.922,1.741]	[-0.482,2.180]
var(e.donation_rel)	0.284***	0.248***	0.260***
	[0.233,0.334]	[0.204,0.292]	[0.214,0.306]
Class FE	No	Yes	Yes
N	592	588	592
p: HIGH = LOW	0.064	0.056	0.128
p: HYPO LOW = LOW	0.374	0.319	0.534
p: HYPO HIGH = HIGH	0.396	0.785	0.971

95% confidence intervals in brackets

* $p < 0.10$, ** $p < 0.05$, *** $p < 0.01$

B.5 Treatment Effects by Grade

We performed an age-specific analysis by splitting the sample by grade. The regression results are presented in this sub-section. The grade is not a perfect delineation between age groups, but an approximation. In each grade, some students have repeated or skipped a grade. However, since very few students are affected by this, we chose to split by grade when analyzing the age-specific effects.

Table B.6: Regression Results - Empirical Expectation by Grade

	(1)	(2)	(3)
	Grade 8	Grade 9	Grade 10
HIGH	0.0537	0.0354	0.0225
	[-0.013,0.121]	[-0.041,0.112]	[-0.047,0.092]
LOW	-0.123***	-0.0815**	-0.124***
	[-0.191,-0.055]	[-0.156,-0.007]	[-0.193,-0.055]
HYPO-LOW	-0.0447	-0.0679	-0.0190
	[-0.137,0.047]	[-0.170,0.035]	[-0.108,0.070]
HYPO-HIGH	0.000804	0.0747	0.0204
	[-0.088,0.090]	[-0.037,0.187]	[-0.074,0.115]
Female	0.0370	0.0689**	0.0955***
	[-0.017,0.091]	[0.009,0.128]	[0.044,0.147]
For. Language: Yes	-0.0588*	-0.0335	-0.0559*
	[-0.118,0.001]	[-0.100,0.033]	[-0.116,0.004]
School B	-0.0354	-0.208***	-0.00968
	[-0.133,0.062]	[-0.334,-0.082]	[-0.117,0.098]
INS Scale	0.000902	-0.0177	0.00278
	[-0.020,0.021]	[-0.043,0.007]	[-0.021,0.027]
Part. FFF protests	-0.0430	0.0274	0.0334
	[-0.095,0.009]	[-0.037,0.092]	[-0.028,0.095]
CFB Scale	0.00576	-0.00202	0.00789
	[-0.009,0.021]	[-0.019,0.015]	[-0.007,0.023]
Constant	0.560***	0.688***	0.550***
	[0.426,0.695]	[0.539,0.836]	[0.412,0.688]
var(e.belief_rel)	0.0269***	0.0338***	0.0341***
	[0.021,0.032]	[0.027,0.041]	[0.028,0.041]
Class FE	Yes	Yes	Yes
N	186	181	224
p: HIGH = LOW	0.000	0.003	0.000
p: HYPO LOW = LOW	0.089	0.789	0.021
p: HYPO HIGH = HIGH	0.233	0.483	0.966

95% confidence intervals in brackets

* $p < 0.10$, ** $p < 0.05$, *** $p < 0.01$

Table B.7: Regression Results - Normative Expectation Small Donations (0-2€) by Grade

	(1)	(2)	(3)
	Grade 8	Grade 9	Grade 10
HIGH	0.119	-0.197	0.205
	[-0.188,0.426]	[-0.523,0.129]	[-0.142,0.552]
LOW	0.531***	0.0682	0.114
	[0.214,0.847]	[-0.251,0.388]	[-0.235,0.462]
HYPO-LOW	0.244	0.117	0.895***
	[-0.174,0.661]	[-0.315,0.548]	[0.470,1.320]
HYPO-HIGH	-0.152	-0.281	0.372
	[-0.576,0.272]	[-0.806,0.243]	[-0.118,0.862]
Female	-0.0248	-0.234*	-0.553***
	[-0.274,0.225]	[-0.491,0.023]	[-0.809,-0.296]
For. Language: Yes	0.249*	0.308**	0.324**
	[-0.023,0.520]	[0.014,0.601]	[0.025,0.623]
School B	0.128	0.195	-0.345
	[-0.319,0.574]	[-0.365,0.755]	[-0.886,0.196]
INS Scale	0.00972	-0.0790	-0.0337
	[-0.085,0.104]	[-0.185,0.027]	[-0.157,0.089]
Part. FFF protests	0.269**	0.0128	-0.0925
	[0.030,0.509]	[-0.265,0.290]	[-0.403,0.218]
CFB Scale	-0.0169	-0.00940	-0.0216
	[-0.087,0.053]	[-0.083,0.064]	[-0.100,0.057]
Constant	-1.932***	-0.985***	-0.985***
	[-2.545,-1.319]	[-1.620,-0.351]	[-1.689,-0.281]
var(e.sar02)	0.472***	0.587***	0.715***
	[0.358,0.586]	[0.450,0.723]	[0.543,0.887]
Class FE	Yes	Yes	Yes
N	159	173	211
p: HIGH = LOW	0.010	0.112	0.601
p: HYPO LOW = LOW	0.172	0.821	0.000
p: HYPO HIGH = HIGH	0.200	0.750	0.495

95% confidence intervals in brackets

* $p < 0.10$, ** $p < 0.05$, *** $p < 0.01$

Table B.8: Regression Results - Normative Expectation Medium Donations (3-5€) by Grade

	(1) Grade 8	(2) Grade 9	(3) Grade 10
HIGH	0.0176	-0.265	0.104
	[-0.268,0.304]	[-0.582,0.052]	[-0.204,0.413]
LOW	0.406***	-0.123	0.0598
	[0.109,0.703]	[-0.438,0.192]	[-0.247,0.366]
HYPO-LOW	0.260	-0.0219	0.440**
	[-0.130,0.650]	[-0.449,0.406]	[0.046,0.834]
HYPO-HIGH	-0.0393	-0.445*	0.167
	[-0.427,0.349]	[-0.960,0.070]	[-0.307,0.640]
Female	-0.0890	-0.157	-0.0730
	[-0.322,0.144]	[-0.410,0.095]	[-0.308,0.162]
For. Language: Yes	0.323**	0.170	0.143
	[0.066,0.580]	[-0.125,0.466]	[-0.128,0.415]
School B	0.259	0.381	-0.0353
	[-0.143,0.662]	[-0.166,0.929]	[-0.532,0.461]
INS Scale	0.0455	0.0620	0.0308
	[-0.044,0.135]	[-0.043,0.168]	[-0.080,0.142]
Part. FFF protests	0.314***	-0.177	-0.154
	[0.091,0.537]	[-0.446,0.093]	[-0.436,0.127]
CFB Scale	-0.0214	0.0120	-0.0536
	[-0.087,0.044]	[-0.060,0.084]	[-0.124,0.017]
Constant	-0.0589	0.450	0.481
	[-0.629,0.511]	[-0.170,1.069]	[-0.150,1.113]
var(e.sar35)	0.411***	0.558***	0.656***
	[0.315,0.507]	[0.432,0.684]	[0.525,0.787]
Class FE	Yes	Yes	Yes
N	156	168	210
p: HIGH = LOW	0.009	0.384	0.778
p: HYPO LOW = LOW	0.458	0.636	0.059
p: HYPO HIGH = HIGH	0.769	0.488	0.795

95% confidence intervals in brackets

* $p < 0.10$, ** $p < 0.05$, *** $p < 0.01$

Table B.9: Regression Results - Normative Expectation High Donations (6-8€) by Grade

	(1) Grade 8	(2) Grade 9	(3) Grade 10
HIGH	0.369	-0.237	0.184
	[-0.099,0.838]	[-0.747,0.272]	[-0.171,0.540]
LOW	0.383	-0.149	0.137
	[-0.105,0.871]	[-0.666,0.367]	[-0.222,0.495]
HYPO-LOW	1.201**	0.323	0.0453
	[0.226,2.177]	[-0.451,1.096]	[-0.415,0.505]
HYPO-HIGH	0.420	-0.425	0.541*
	[-0.230,1.070]	[-1.237,0.387]	[-0.077,1.158]
Female	-0.0753	-0.0837	-0.0826
	[-0.474,0.323]	[-0.493,0.326]	[-0.361,0.196]
For. Language: Yes	0.160	-0.0298	0.0962
	[-0.294,0.614]	[-0.526,0.466]	[-0.229,0.421]
School B	0.377	0.0840	0.0591
	[-0.296,1.051]	[-0.823,0.991]	[-0.542,0.660]
INS Scale	0.0822	0.0666	0.0250
	[-0.079,0.243]	[-0.116,0.250]	[-0.105,0.155]
Part. FFF protests	0.248	-0.457**	-0.0620
	[-0.143,0.638]	[-0.900,-0.015]	[-0.389,0.265]
CFB Scale	-0.0195	0.0347	-0.0632
	[-0.127,0.088]	[-0.085,0.154]	[-0.146,0.020]
Constant	1.380***	2.499***	2.369***
	[0.408,2.351]	[1.448,3.550]	[1.599,3.139]
var(e.sar68)	0.776***	1.046***	0.665***
	[0.447,1.106]	[0.629,1.462]	[0.442,0.888]
Class FE	Yes	Yes	Yes
N	157	169	211
p: HIGH = LOW	0.957	0.732	0.796
p: HYPO LOW = LOW	0.099	0.223	0.695
p: HYPO HIGH = HIGH	0.879	0.645	0.251

95% confidence intervals in brackets

* $p < 0.10$, ** $p < 0.05$, *** $p < 0.01$

Table B.10: Regression Results - Donations by Grade

	(1)<br>Grade 8	(2)<br>Grade 9	(3)<br>Grade 10
HIGH	0.142*	0.00540	-0.0283
	[-0.007,0.290]	[-0.226,0.237]	[-0.323,0.266]
LOW	-0.151**	-0.0642	0.0241
	[-0.300,-0.002]	[-0.291,0.162]	[-0.277,0.325]
HYPO-LOW	-0.0535	-0.0860	0.244
	[-0.254,0.147]	[-0.402,0.230]	[-0.163,0.650]
HYPO-HIGH	-0.0123	0.206	-0.0342
	[-0.209,0.185]	[-0.146,0.559]	[-0.429,0.361]
Female	0.121**	0.236**	0.429***
	[0.001,0.241]	[0.056,0.415]	[0.199,0.658]
For. Language: Yes	-0.225***	-0.319***	-0.355***
	[-0.356,-0.094]	[-0.520,-0.118]	[-0.606,-0.105]
School B	-0.121	-0.350*	-0.145
	[-0.339,0.097]	[-0.732,0.032]	[-0.612,0.323]
INS Scale	0.0315	0.0378	0.0299
	[-0.014,0.077]	[-0.039,0.114]	[-0.076,0.136]
Part. FFF protests	0.0514	0.118	0.0356
	[-0.063,0.166]	[-0.073,0.309]	[-0.239,0.311]
CFB Scale	0.0271	0.0306	0.133***
	[-0.006,0.061]	[-0.022,0.083]	[0.064,0.202]
Constant	0.394***	0.631***	0.229
	[0.096,0.692]	[0.170,1.092]	[-0.365,0.822]
var(e.donation_rel)	0.123***	0.264***	0.458***
	[0.092,0.155]	[0.179,0.350]	[0.288,0.628]
Class FE	Yes	Yes	Yes
N	186	182	224
p: HIGH = LOW	0.000	0.546	0.737
p: HYPO LOW = LOW	0.334	0.888	0.294
p: HYPO HIGH = HIGH	0.120	0.251	0.977

95% confidence intervals in brackets

* $p < 0.10$, ** $p < 0.05$, *** $p < 0.01$

C Robustness Check: Hypo Treatments to control for Demand and Anchoring Effects

C.1 Descriptives

As robustness analysis we control for potential confounding effects in the HIGH and LOW treatment due to the pure provisioning of donation amounts. In particular, experimenter demand and anchoring effects may be triggered due to the listing of specific donation amounts, irrespective whether these donations were made by a former survey participant (i.e. another student from a local high school).

In HYPO-HIGH and HYPO-LOW, participants received information about the same donation amounts as in HIGH and LOW respectively. However, the donations were explicitly labelled as hypothetical. We believe that these hypothetical donations induce anchoring and demand effects at least as much as the HIGH and LOW treatments. Since the donations are labelled as hypothetical, participants may question even more why these specific donations amounts were listed. This could induce even stronger experimenter demand effects than in the LOW and HIGH conditions. Comparing donations between HYPO-HIGH and HIGH and between HYPO-LOW and LOW hence provides conservative, lower bound estimates for the treatment effects.

All regression models presented in Section B also estimate the effect of the two HYPO treatments relative to CONTROL. For each regression model, we present at the bottom of the table Wald tests that test whether the respective HYPO treatment coefficient is statistically significantly different from the main treatment coefficient.

Table C.1: Donation and Empirical Expectation by Treatment

	**Empirical Expectation**			**Donation**		
	Mean	SD	Median	Mean	SD	Median
**Treatment**						
CONTROL (n=164)	0.56	0.21	0.50	0.64	0.34	0.63
HIGH (n=168)	0.60	0.21	0.63	0.63	0.35	0.63
LOW (n=159)	0.46	0.20	0.44	0.59	0.37	0.50
HYPO-LOW (n=70)	0.51	0.23	0.50	0.61	0.37	0.63
HYPO-HIGH (n=67)	0.57	0.21	0.50	0.63	0.34	0.63
Total (n=628)	0.54	0.21	0.50	0.62	0.35	0.63

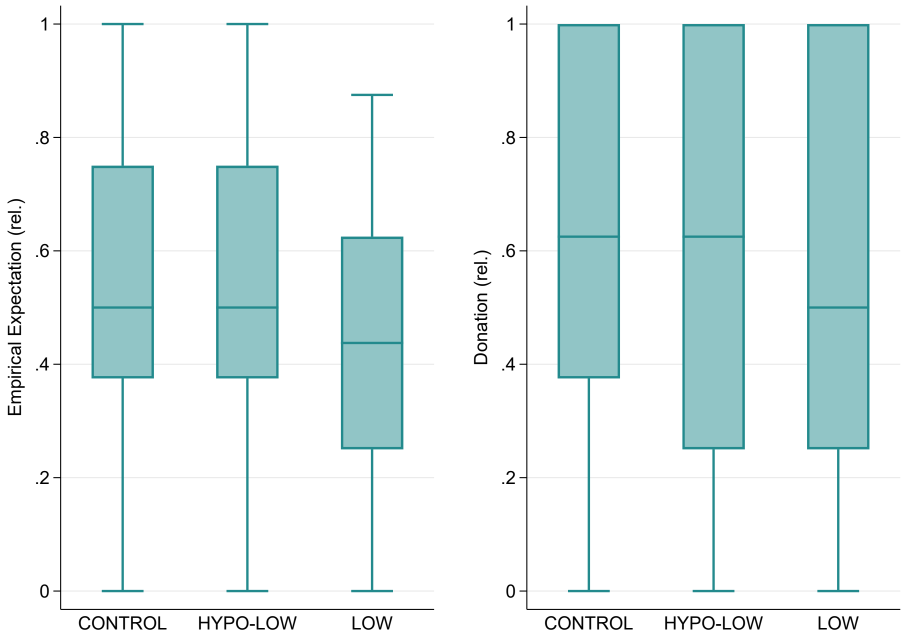

Figure C.1: Boxplot - LOW vs HYPO-LOW

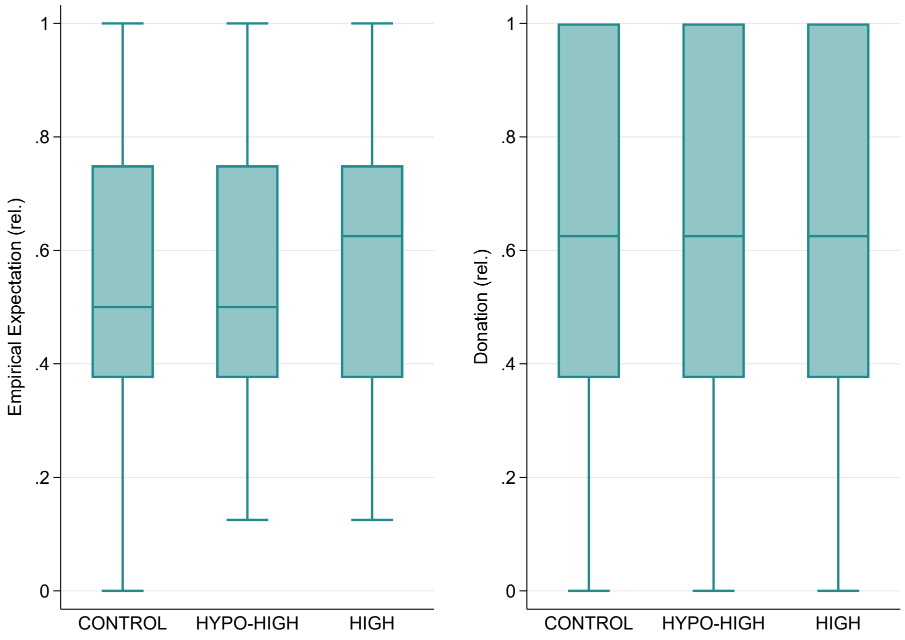

Figure C.2: Boxplot - HIGH vs HYPO-HIGH

D Experimental Material

D.1 Pictures of the Implementation

Figure D.1: Randomization of Treatments via Seating Plan

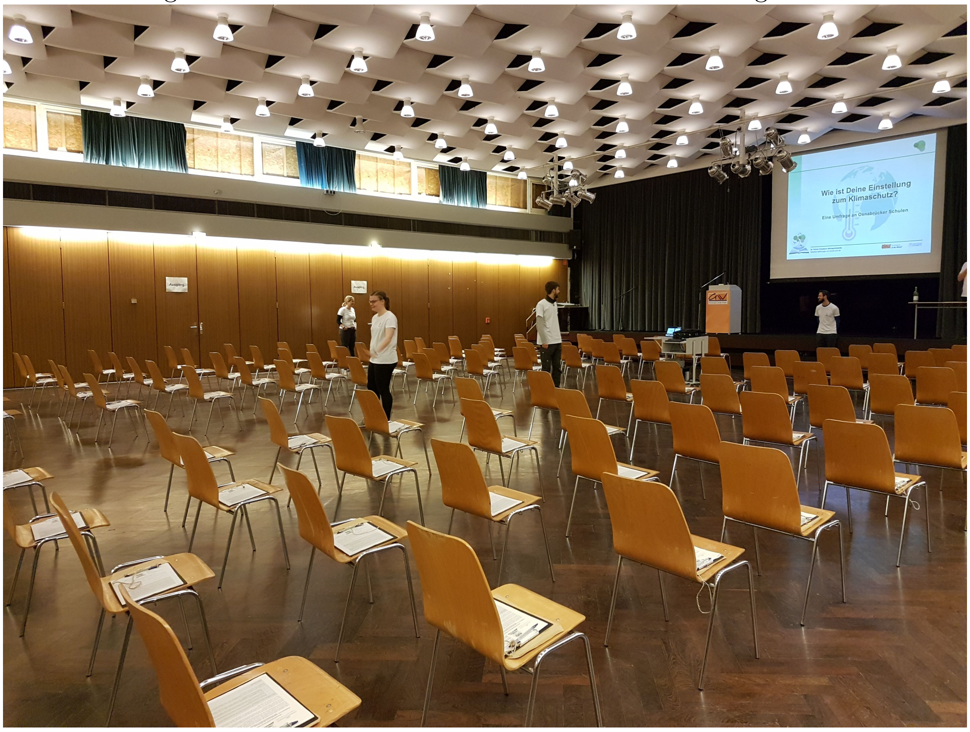

Figure D.2: Seating in Assembly Hall of School A

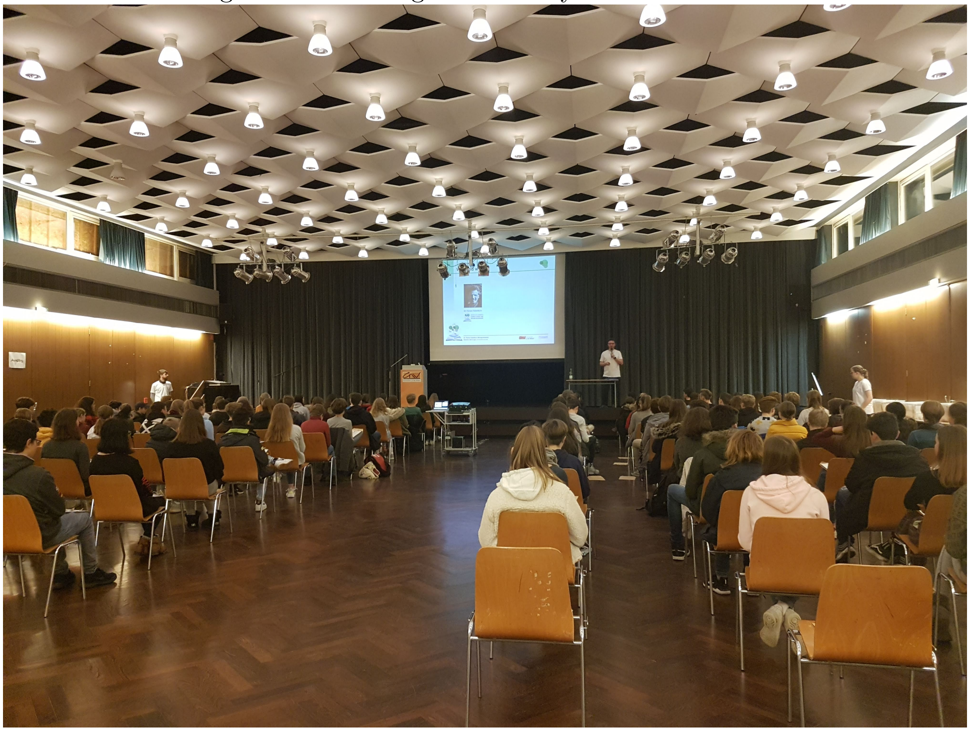

Figure D.3: Seating in Assembly Hall of School B

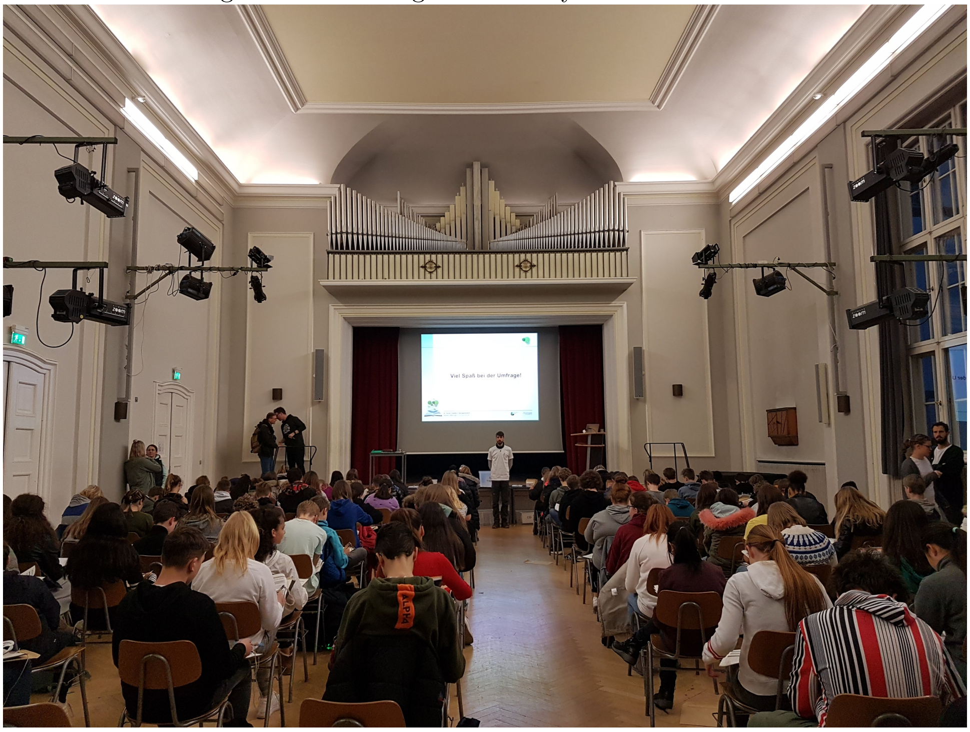

D.2 Questionnaire and Instructions

Below, we include a translated version of the original questionnaire used for the data collection. The questionnaire consists of three parts:

1. The first part includes only the donation decision. At the beginning, the benefiting organization and the particular use of donations is presented. In the section "How do you know that the money is really donated" participants learned how they could verify that their donation was indeed transferred to the beneficiary. Besides the original donation receipts, it is explained that a detailed account of all individual donations will be made accessible online. With the unique, but anonymous ID, participants can verify whether their donation was correctly listed and transferred. Due to logistic reasons, it was not possible to receive a donation receipt for each single donation amount. Furthermore, we used the detailed account of individual donations to introduce our treatments. In the LOW and HIGH treatment, three exemplary donation amounts were presented. These presented donations were real and made by students from another high school in the same city, where we run our pre-test. In the two HYPO treatments, the presented donations were explicitly labelled as hypothetical. The variations between treatments are included in the translated questionnaire below in brackets.
2. The second part of the survey includes the incentivized elicitation of the empirical expectation. The instructions were the same for all respondents.
3. The third part includes the incentivized elicitation of the normative expectation. Again, there were no differences between treatments. This part also includes a number of questions on the socio-economic background, pro-environmental behaviour and attitudes.

4017040860

ENGLISH TRANSLATION:

What is your attitude towards climate protection?

A survey in the schools of Osnabrück

Part 1

Brief information about the survey

As said in the presentation, you will receive 8 euros. You can decide what to do with this money. Further down on the page, you will be able to choose how much of the 8 euros will be paid out to you and how much will be donated to the organization *myclimate* to offset CO2 emissions.

What will your donation be used for?

myclimate is a non-profit organization which offers individuals the opportunity to offset CO2 emissions. This means that donations are used to support projects worldwide that reduce CO2 emissions. Such projects promote, for example, the use of renewable energies or the reforestation of forests. Every euro donated can therefore reduce a certain amount of CO2 emissions.

The following information on the use of donations is provided on the myclimate website:

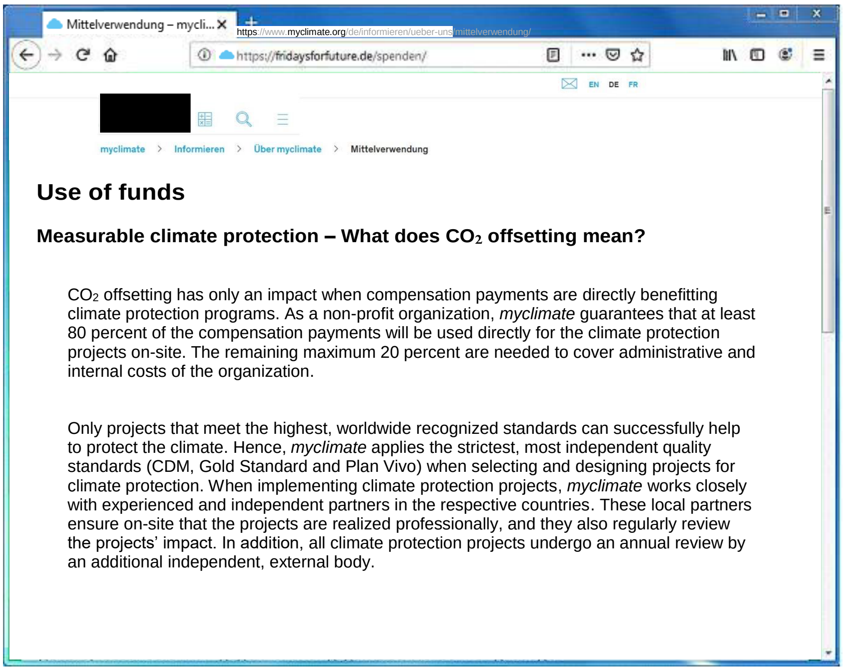
Mittelverwendung – mycli...
https://www.myclimate.org/de/informieren/ueber-uns/mittelverwendung/
https://fridaysforfuture.de/spenden/
EN DE FR
[redacted]
myclimate > Informieren > Über myclimate > Mittelverwendung

Use of funds

Measurable climate protection – What does CO_2 offsetting mean?

CO_2 offsetting has only an impact when compensation payments are directly benefitting climate protection programs. As a non-profit organization, *myclimate* guarantees that at least 80 percent of the compensation payments will be used directly for the climate protection projects on-site. The remaining maximum 20 percent are needed to cover administrative and internal costs of the organization.

Only projects that meet the highest, worldwide recognized standards can successfully help to protect the climate. Hence, *myclimate* applies the strictest, most independent quality standards (CDM, Gold Standard and Plan Vivo) when selecting and designing projects for climate protection. When implementing climate protection projects, *myclimate* works closely with experienced and independent partners in the respective countries. These local partners ensure on-site that the projects are realized professionally, and they also regularly review the projects' impact. In addition, all climate protection projects undergo an annual review by an additional independent, external body.

Mittelverwendung – mycli... ×

https://www.myclimate.org/de/informieren/ueber-uns/mittelverwendung/

EN DE FR

[redacted]

myclimate > Informieren > Über myclimate > Mittelverwendung

Process – What happens with the money?

myclimate supports climate protection projects on a service-specific basis. Only reported, actual emission reductions over a longer contract period of 7 to 14 years are accounted for in energy projects. The compensation payments will be paid out to the supported projects once the reductions are realized. The actual contribution amount depends on the volume of climate-impacting emissions reduced. Forestry projects are in this regard somewhat different since their timelines naturally stretches over a longer span of 30 to 50 years.

myclimate guarantees its customers that the bought emission reductions (= compensation donations) will be in at the latest two years realized and will be retired after a maximum of three years. In forest projects, carbon storage occurs within 10 to 30 years, depending on the project region and tree species. To achieve the reduction in forest projects several special and comprehensive mechanisms are in place.

Every Euro donated currently reduces emissions by an equivalent of 42 kg CO_2 emissions. This amount of CO_2 can be illustrated by the following examples:

- An average new car emits this amount of CO_2 over a distance of approximately 237 km.
- The production of Apple's latest iPhone causes approximately 57 kg of CO_2 emissions per device.
- The production of a white long-sleeve cotton shirt causes approximately 4.3 kg of CO_2 emissions during production.

How do you know that the money is really donated?

So that you can later check whether your donation has actually been transferred to *myclimate*, we will publish the donation receipt on our homepage within the next two weeks. This confirmation will show the total amount donated by all your schoolmates.

You can find this confirmation at: www…….

The picture below shows an example of what the donation confirmation will look like.

At the end of the survey you will receive a piece of paper with your personal code, this web link and a QR code that you can take home.

[redacted]

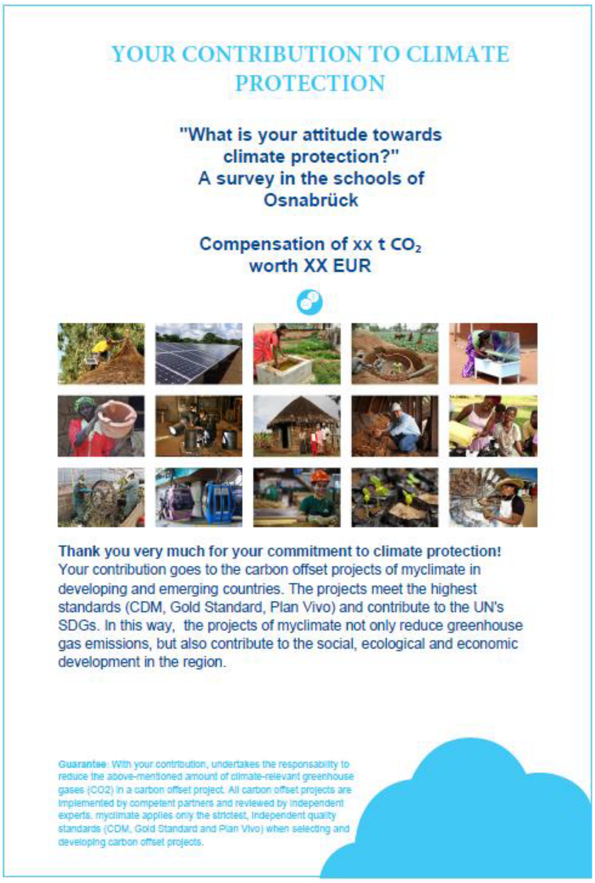

YOUR CONTRIBUTION TO CLIMATE PROTECTION

"What is your attitude towards climate protection?"
A survey in the schools of Osnabrück

Compensation of xx t CO_2
worth XX EUR

Thank you very much for your commitment to climate protection!
Your contribution goes to the carbon offset projects of myclimate in developing and emerging countries. The projects meet the highest standards (CDM, Gold Standard, Plan Vivo) and contribute to the UN's SDGs. In this way, the projects of myclimate not only reduce greenhouse gas emissions, but also contribute to the social, ecological and economic development in the region.

Guarantee: With your contribution, undertakes the responsability to reduce the above-mentioned amount of climate-relevant greenhouse gases (CO2) in a carbon offset project. All carbon offset projects are implemented by competent partners and reviewed by independent experts. myclimate applies only the strictest, independent quality standards (CDM, Gold Standard and Plan Vivo) when selecting and developing carbon offset projects.

8725040860

How do you know that the money is really donated?

In addition to the certificate from *myclimate*, we will put a list online with the individual donations, which will list the personal code and the respective donation amount in a table. The screenshot shows how the donations will be displayed.

What is your attitude towards climate protection?
A survey in the schools of Osnabrück

List of individual donations

Date of the survey: 17.12.2019

Code	Donation Amount in €uro
GA-089	X €<br>[HIGH/ HYPO-HIGH: 8 €]<br>[LOW/ HYPO-LOW: 0 €]
HB-007	X €<br>[HIGH/ HYPO-HIGH: 6 €]<br>[LOW/ HYPO-LOW: 2 €]
GC-127	X €<br>[HIGH/ HYPO-HIGH: 7 €]<br>[LOW/ HYPO-LOW: 1 €]

[LOW/ HIGH: In this case, you see the donations of three students of your age from another high school in Osnabrück, who have previously participated in this study.]

[HYPO-: As an example, we show you here three fictive donation amounts.]

- [LOW/ HIGH: The student with the code GA-089 donated [High: 8€ / LOW: 0€]. This means he or she received [HIGH: 0€ / LOW: 8€] as a payout.]
  [HYPO-: Imagine the student with the code GA-089 would have donated [HIGH: 8€ / LOW: 0€]. This means he or she would receive [HIGH: 0€ / LOW: 8€] as a payout
- [LOW/ HIGH: The student with the code HB-007 donated [High: 6€ / LOW: 2€]. This means he or she received [HIGH: 2€ / LOW: 6€] as a payout.]
  [HYPO-: Imagine the student with the code HB-007 would have donated [HIGH: 6€ / LOW: 2€]. This means he or she would receive [HIGH: 2€ / Low: 6€] as a payout.]
- [LOW/ HIGH: The student with the code GC-127 donated [High: 7€ / LOW: 1€]. This means he or she received [HIGH: 1€ / LOW: 7€] as a payout.
  [HYPO-: Imagine the student with the code GC-127 would have donated [HIGH: 7€ / LOW: 1€]. This means he or she would receive [HIGH: 1€ / LOW: 7€] as a payout

8725040860

In the original version on the bottom of the previous page.

**Your donation decision**									
I would like to donate the following amount to *myclimate*: *(Please tick only once.)*									
myclimate receives...	0€	1€	2€	3€	4€	5€	6€	7€	8€
	☐	☐	☐	☐	☐	☐	☐	☐	☐
I receive...	8€	7€	6€	5€	4€	3€	2€	1€	0€

3113040866

What is your attitude towards climate protection?

A survey in the schools of Osnabrück

Part 5

1674040866

Your classmates and their donation decisions

Here you have now the possibility to **additionally receive 1 Euro**. Whether you get it depends on your own decision and the decision of your schoolmates.

Please estimate below how many euros your classmates from your grade have donated to *myclimate* on average.

Once we have collected all questionnaires, we will calculate the average of all donations. If you correctly estimated the average (rounded to a full Euro amount), you will receive additionally 1 Euro.

I estimate that, on average, my schoolmates from my grade donated the following amount to *myclimate*: *(Please tick only once.)*								
0€	1€	2€	3€	4€	5€	6€	7€	8€
☐	☐	☐	☐	☐	☐	☐	☐	☐

3787040863

What is your attitude towards climate protection?

A survey in the schools of Osnabrück

Part 3

0988040863

Social Appropriateness of Donations

We have another estimation task for you, also here you can receive **1 Euro**. Again this depends on your decision and the decision of your schoolmates.

In the following, we will ask you to what extent the individual donation amounts are considered socially appropriate. **Socially appropriate behavior** is behavior that the majority of people consider to be ethically acceptable. If the majority of people is annoyed by a certain behavior, then the behavior is considered socially inappropriate.

For every possible donation between 0 and 8 euros, you shall indicate in the following table how socially appropriate your schoolmates consider this donation amount. At the end of the survey, we will compare your answers with those of your schoolmates. Which estimates we will compare will be randomly decided later in front of you. If you have given the same answer in this estimation task as most of your schoolmates, you will receive additionally 1 Euro.

How socially appropriate do the students from your grade think it is to donate the following amounts (of the 8 Euros) to *myclimate*?

(Please tick in the table for each possible donation decision, how socially appropriate your schoolmates consider this donation amount to be).

Donation	Very socially inappropriate	Somewhat socially inappropriate	Somewhat socially appropriate	Very socially appropriate
0€	☐	☐	☐	☐
1€	☐	☐	☐	☐
2€	☐	☐	☐	☐
3€	☐	☐	☐	☐
4€	☐	☐	☐	☐
5€	☐	☐	☐	☐
6€	☐	☐	☐	☐
7€	☐	☐	☐	☐
8€	☐	☐	☐	☐

7760040868

[ONLY (HYPO-) HIGH and LOW:] **In Part 1 you were shown how a donation receipt will look like. In the donation receipt there was a table in which three donations were listed.**

Please cross the correct answer.

- ☐ These donations were made up.
- ☐ These donations were actually made by students from my school.
- ☐ These donations were actually made by students from another high school in Osnabrück.
- ☐ These donations were made by adults.
- ☐ I don't know.

Brief information on the last part of the survey

This part of the questionnaire is about your attitudes towards climate change and the demonstrations of the *Fridays for Future* movement, which you may have participated in.

First, a few questions about yourself

Age: ____________ **Gender**: ☐ male ☐ female ☐ diverse

My school is: ______________________________

What grade are you in? ____ ____

Example: 8 b

How long have you been at your school? ____________ years

In what part of town do you live? ______________________________

Zip Code: ______________________________

Are most of your close friends from your class? ☐ Yes ☐ No

If not, where do most of your close friends go to school? *(multiple choice possible)*

- ☐ Parallel class
- ☐ Other grade on my school
- ☐ Other school(s)

4493040868

Is another language than German spoken at home? ☐ Yes ☐ No

If so, which one(s)? ______________________________

A few questions about your volunteer work

Did you take part in the *Fridays for Future* protests for climate protection this year? ☐ Yes ☐ No

You only need to answer these questions if you crossed **"Yes"!**

How often? ☐ Once ☐ More than once

Why did you participate?
(multiple answers possible)

☐ I am concerned about climate change.

☐ My engagement can make a difference.

☐ My friends have participated.

☐ Possibility not to go to school.

☐ Other reason(s): ______________________________

You only have to answer these questions if you crossed **"No"**!

Why have you not participated in the *Fridays for Future* protests for climate protection? *(multiple choice possible)*

☐ I am not concerned about climate change.

☐ My engagement cannot change anything.

☐ My friends do not go to the protests and I do not want to go alone.

☐ I do not want to violate the law of mandatory school attendance.

☐ My performance at school may worsen if I miss classes.

☐ I fear negative consequences from the teachers if I do not show up to class and participate in the protests.

☐ Other reason(s): ______________________________

Protests and compulsory school education

	very inappropriate	inappropriate	neutral	appropriate	very appropriate
How socially appropriate do **you** think it is for students to protest for climate protection during school hours?	☐	☐	☐	☐	☐
How socially appropriate do you think it is for **your parents** that students protest for climate protection during school hours?	☐	☐	☐	☐	☐
How socially appropriate do you think it is for **your teachers** that students protest for climate protection during school hours?	☐	☐	☐	☐	☐

Reactions to protests during school hours

Imagine you would go to the next *Fridays for Future* protest during school hours.

What would be **your parents'** reaction? *(Only one answer please)*

- ☐ They would approve my decision and support me in case I would get in trouble at school.
- ☐ They would approve my decision.
- ☐ They would not approve my decision.
- ☐ They would not approve my decision and would have a serious conversation with me.
- ☐ They would not approve my decision and would threaten me with consequences (e.g. reduction of my pocket money or house arrest).

What would be your **teachers'** reaction? *(Only one answer please)*

- ☐ They would approve my decision and support me when needed.
- ☐ They would approve my decision.
- ☐ They would not approve my decision.
- ☐ They would not approve my decision and would have a serious conversation with me.
- ☐ They would not approve my decision and would threaten me with consequences (e.g. bad grades due to unexcused absence).

What are you doing to protect the climate?
(multiple answers possible)

- ☐ I participate in the *Fridays for Future*-protests.
- ☐ I am involved in the organization of the *Fridays for Future*-movement.
- ☐ I eat little or no meat.
- ☐ I try not to fly with the plane on vacations and also try to convince my parents not to do so either.
- ☐ I try to avoid using electricity when it is not needed.
- ☐ I try not to take long showers and not so often.
- ☐ I try to ride my bike often.
- ☐ I try to get my parents to buy more seasonal and regional products.
- ☐ I try to talk my parents into buying energy-efficient household appliances.
- ☐ I am convincing my parents to switch to green energy.
- ☐ I try to avoid generating (plastic) garbage.
- ☐ I do nothing to protect the climate.

Your relationship to nature

How connected are you with nature?

Please tick the box of the picture that best reflects your connection with nature.

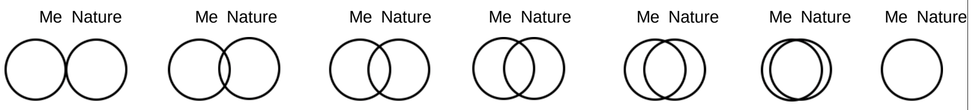

☐ ☐ ☐ ☐ ☐ ☐ ☐

A few final questions about your participation in the *Fridays for Future* protests

Participating in the *Fridays for Future* demonstrations is for me...

	⊖ 1	2	3	4	5	⊕ 6	7	
very bad	☐	☐	☐	☐	☐	☐	☐	very good
very negative	☐	☐	☐	☐	☐	☐	☐	very positive
very stupid	☐	☐	☐	☐	☐	☐	☐	very smart
very boring	☐	☐	☐	☐	☐	☐	☐	very exciting
very disadvantageous	☐	☐	☐	☐	☐	☐	☐	very advantageous
very worthless	☐	☐	☐	☐	☐	☐	☐	very valuable
very unpleasant	☐	☐	☐	☐	☐	☐	☐	very pleasant

1019040869

	very unlikely 1	2	3	4	5	6	very likely 7
**People who are important to me** think I should participate in the *Fridays for Future* protests.	☐	☐	☐	☐	☐	☐	☐
**My family** thinks I should participate in the *Fridays for Future* protests.	☐	☐	☐	☐	☐	☐	☐
**My friends** think I should participate in *Fridays for Future* protests.	☐	☐	☐	☐	☐	☐	☐
**My teachers** think I should participate in *Fridays for Future* protests.	☐	☐	☐	☐	☐	☐	☐

	very incorrect 1	2	3	4	5	6	very correct 7
I plan to participate in the *Fridays for Future* protests.	☐	☐	☐	☐	☐	☐	☐
I want to try to participate in the *Fridays for Future* protests.	☐	☐	☐	☐	☐	☐	☐
I certainly do want to participate in the *Fridays for Future* protests.	☐	☐	☐	☐	☐	☐	☐
I am completely sure that I will participate in the *Fridays for Future* protests.	☐	☐	☐	☐	☐	☐	☐

	very unlikely 1	2	3	4	5	6	very likely 7
Participating in *the Fridays for Future* protests is for me…	☐	☐	☐	☐	☐	☐	☐

	very unlikely 1	2	3	4	5	6	very likely 7
If I wanted to, it would be easy for me to participate in the *Fridays for Future* protests.	☐	☐	☐	☐	☐	☐	☐

	very incorrect 1	2	3	4	5	6	very correct 7
The decision to participate in the *Fridays for Future* protests is completely in my control.	☐	☐	☐	☐	☐	☐	☐

1019040869

	very difficult 1	2	3	4	5	6	very easy 7
For me, participating in the *Fridays for Future* protests is…	☐	☐	☐	☐	☐	☐	☐

Further comments

If you would like to say something else about the survey or the topic, you can do it here:

Thank you very much for your participation!
